# The influence of early life socialisation on cognition in the domestic pig (*Sus scrofa domestica*)

**DOI:** 10.1038/s41598-020-76110-5

**Published:** 2020-11-05

**Authors:** Jennifer E. Weller, Simon P. Turner, Agnieszka Futro, Jo Donbavand, Mark Brims, Gareth Arnott

**Affiliations:** 1 Institute for Global Food Security, School of Biological Sciences, Queens University Belfast, Belfast, UK; 2 Department of Comparative Pathobiology, Purdue University, West Lafayette, IN USA; 3 Animal Behaviour & Welfare, Scotland’s Rural College (SRUC), Edinburgh, UK

**Keywords:** Zoology, Animal behaviour

## Abstract

Previously, the benefits of early-life socialisation on later-life social development have been reported in pigs. Here we investigated the effect of pre-weaning socialisation on the later-life cognitive ability of pigs using a range of techniques. Pre-weaning, 101 piglets had access to a neighbouring pen from ~ 15 days of age and interacted with non-littermates (socialised). An additional 89 piglets remained isolated within their home pen (controls). After weaning, 100 individuals were selected for a range of cognitive tests including a food reward T-maze test, reversal learning T-maze test, a social preference T-maze test, and a puzzle box test. Performance during the food reward test was not influenced by treatment. Treatment effected improvement over the course of the reversal learning test, with controls showing a significant decrease in trial duration after the first two trials. During the social preference test, socialised pigs spent significantly more time in the presence of larger stimulus pigs than controls and were quicker to leave the middle of the maze, suggesting improved social skills. Neither sex nor treatment was observed to influence pig’s ability to solve the puzzle box. Thus, overall, evidence from the social preference test suggests an effect of pre-weaning socialisation on aspects of social cognitive development.

## Introduction

Broadly speaking, the term ‘animal cognition’ refers to the collection of processes through which an animal obtains, retains, and decides to act upon information gathered about the world around them^1^. Such processes, including memory, learning, problem solving, and communication, have been studied in an extensive range of species^2^, including the domestic pig, *Sus scrofa domestica* [reviewed in^3–5^]. Domesticated animals kept in a commercial setting are often unintentionally exposed to memory and learning challenges^6,7^ that can impact welfare. Memory and learning challenges often include the memorization of feeding routines and environmental aspects, the use of new or unfamiliar feeding apparatus, and the recognition of cues signalling husbandry practices. If pigs are not equipped with the cognitive abilities required to cope with these challenges, their welfare will likely be negatively impacted^3^.

Sneddon et al.^8^ suggested that the current, low-quality environments associated with modern pig production systems may result in cognitive impairment, alongside the development of other behavioural and physical problems. Understanding species-specific cognition and how it can be manipulated through environmental factors, can therefore be used to facilitate the improvement of housing conditions, handling routines, and stock management^7,9,10^.

Animal welfare can also be indirectly influenced by the public’s perception of animal intelligence^4^. For example, pigs are generally seen as being ‘intelligent’ animals ^5^, and many consumers consider poor welfare resulting from farming related practices to be immoral^11^. This can result in an increased demand for ‘high welfare’ products, leading to positive changes in farmer attitudes and improved legislation concerning the welfare of domestic livestock.

One particularly common welfare concern is the frequent mixing of unfamiliar pigs on commercial farms^12–14^. Pigs are a highly social species^4,15^ that use aggression to establish a new dominance hierarchy in the presence of unfamiliar individuals^16–18^. Furthermore, this aggression can be amplified by housing conditions, which often do not provide enough space for individuals to display correct threat and retreat behaviours^19,20^. Typically, piglets are kept isolated within their litter groups for the entirety of the pre-weaning period. However, it has previously been reported that allowing piglets to interact with non-litter mates during this period, termed socialisation, can lead to a reduction in agonistic behaviours later in life^17,18,21–24^. While prior research has considered the effect of socialisation on aggression as a repeatable personality trait^17,25^, few studies have considered what effect early life socialisation has on other aspects of pig behaviour [but see^25,26^].

Multiple studies have shown that the quality of a pig’s early life environment can influence cognition with regards to both learning^8^ and memory^27^. For example, Bolhuis et al*.*^28^ found that environmental enrichment can lead to the improvement of working memory (short-term memory concerned with immediate conscious perception^29^) but not reference memory (longer-term memory concerned with the retention of information over time^29^). Despite this, the effect of the early life social environment on such aspects of cognition have yet to be explored. Previous research has revealed that the early life social environment can influence brain development in a number of species, including the common frog (*Rana temporaira*), laboratory mice (*Mus musculus*), and cooperatively breeding cichlids (*Neolamprologus pulcher*)^30–32^. Furthermore, the social intelligence hypothesis^33^ suggests that group living requires the enhancement of cognitive abilities due to the need to monitor other group members, recognise previously cooperative partners, and maintain multiple relationships with other group members^34^. For example, proxies of social complexity such as the formation of long-term monogamous relationships, social group size, and variation in kinship between group members have been found to correlate with indicators of increased cognitive ability, such as brain size and improved performance in cognitive tasks^35–37^. Therefore, the aim of this study was to investigate the effect of pre-weaning social experience on both social and non-social aspects of cognition in the domestic pig using a range of existing and novel techniques.

Firstly, we tested the operant learning (defined as the performance of a learned response to a stimulus, based on prior associations between the stimulus and either a positive or negative outcome) and working/reference memory of pigs (defined above) that had experienced pre-weaning socialisation (socialised) and those that had not (control). Socialised individuals were expected to learn the location of a food reward sooner than controls and subsequently make fewer entries into the unbaited (incorrect) arm of the maze. Once the location of the reward had been successfully memorised (as indicated by a specified learning criteria), the propensity of pigs to develop inflexible responses to a learned environmental situation was explored using a reversal learning test [see^38,39^]. It was predicted that socialised individuals would be more flexible in their learning and better able to adjust their previously learned responses (referred to as ‘reversal learning’) as the pre-weaning period has been suggested to be an important period for the development of behavioural flexibility^40^. Events experienced during this period are therefore likely to influence an individual’s ability to deal with novel situations later in life. This increased flexibility would allow socialised individuals to find a relocated food reward with greater success than control individuals due to increased experience of unpredictable situations, caused by the presence of non-littermates, pre-weaning.

Secondly, the ability of pigs to obtain and process information about socially relevant conspecifics, referred to as social cognition, was investigated. Previous investigation of social cognition has been focused upon an individual’s ability to recognise related and unrelated conspecifics [reviewed in^41^], store information about others, such as location^42^ or familial relations^43,44^, and discern information about the short-term mental state or knowledge of an another individual^45–47^. Here we investigate if early life socialisation improves an individual’s ability to make assessments about their conspecifics resource holding potential (RHP) using a novel social preference test. RHP refers to an individual’s ability to compete for and retain the resources required for survival and reproduction^48,49^ and is often considered to be represented by an individual’s body size or weight [^50,51^, but see^52^ for review]. Therefore, larger individuals are predicted to be more successful during agonistic encounters. Given this, the mutual assessment hypothesis predicts that if individuals are able to determine RHP, the individual with the lowest RHP should retreat from contests, rather than waste energy engaging in a costly, aggressive interactions they are unlikely to win^52^. However, while cognitive capacity required for individuals to make assessments regarding both their opponents RHP and their own relative RHP has been debated^52–54^, it currently remains unexplored. We predict that if pigs are able to assess the RHP of unfamiliar individuals, they will preferentially aim to retrieve a food reward located near smaller conspecifics compared with a food reward of equal value located near larger conspecifics. Additionally, if early life social experience increases social cognition, we predict that socialised individuals will be quicker and more accurate when making decisions about who to forage near.

Lastly, in order to test innovation, pigs were presented with a sliding-door puzzle box containing a food reward. Innovation, or an individual’s ability to solve either a novel problem or an existing problem with a new solution^55,56^, may have important consequences for survival and reproduction^57^. Here we explore the effect of early life socialisation on innovation, with the hypothesis that individuals experiencing early life socialisation will be quicker and more efficient at ‘solving’ the puzzle box than controls.

It is important to note that previous studies have shown pigs raised in barren environments tend to spend more time performing exploratory behaviours when presented with a novel object or environment^38,58–60^. One potential reason for this is that pigs from barren environments may have an unfulfilled motivation to perform exploratory behaviours^58,59^ such as rooting. Additionally, several studies have observed a positive correlation between exploration and innovation^61,62^ with greater diversity in initial exploration resulting in increased problem-solving success^55^. As such, control individuals may be more motivated to interact with the puzzle-box than those with experience of socialisation, increasing the likelihood of them solving the puzzle accidently (i.e. moving the slider without the intention of accessing the food reward). To account for this, all individuals used in this study performed two novel object tests allowing for a measure of neophobia (hereafter referred to as neophobia score) to be assigned to each pig. This was then included in analyses of behaviours directed towards the puzzle-box.

The overall aim of this paper is to explore the effect that pre-weaning socialisation has on post-weaning performance across a number of tasks testing different elements of cognition. We predict that socialised individuals will be better able to remember the location of a food reward across T-Maze trials and subsequently adapt to a change in location faster than control individuals, representing learning flexibility in addition to improved reference memory. Additionally, we present a new method for testing social cognition that explores an individual’s ability to assess its competitors’ RHP and make foraging decisions based upon collected information. Socialised individuals are predicted to be better able to recognise asymmetries in body size than controls and therefore spend more time foraging near less intimidating pigs in order to minimise the likelihood of feeding displacement. Finally, we predict that despite a potentially increased motivation to perform investigatory behaviours, control pigs will be less innovative than socialised individuals when attempting to access a rewarded puzzle box.

## Methodology

### Ethical note

This study was approved by SRUC’s Animal ethics committee (no. ED AE 34/2017) and adhered to ASAB/ABS guidelines.

### Animals and housing

This study was conducted on 190 individuals (101 males, 89 females) selected from 19 litters born across 2 farrowing events (referred to as batches). All pigs ([large white × landrace] × Duroc) were born between November 2017 and January 2018 at SRUC Easter Howgate pig unit (Roslin, Scotland) and reared in conventional farrowing pens. Males were not castrated and tails/teeth were kept intact. Ten litters underwent a socialisation treatment pre-weaning in which they were able to access one other neighbouring pen through a ~ 35 × 74 cm gap within the pen barrier from ~ 15 days of age (101 piglets). Access to the adjacent pen was allowed until weaning. The remaining nine litters were kept isolated within their home pen (control) as is standard practice on most commercial pig units (89 piglets). Piglets were weaned at four weeks of age by removal of the sow from the home pen. Socialised pigs were once again separated and all pigs were tagged and weighed.

One hundred piglets (52 Socialised, 48 Control) were selected for testing based on the proximity of their body weight to the average body weight of their litter at weaning (litter mean weight at weaning = 8.45 ± SD 1.07 kg; average difference of selected individuals from litter mean = 0.74 ± SD 0.63 kg). Initially two males and two females from each litter were selected where possible, although this was increased to three males and three females for the second batch in order to increase the sample size. Five days after weaning selected pigs were moved to the experimental facilities where they were kept with their siblings in pens measuring ~ 2.15 × 2.15 m. Both water and solid feed pellets were provided ad libitum, in addition to edible straw bedding which was replaced daily. Individuals were allowed to habituate to both their new home pen and human interaction over the course of four days. All pigs were subsequently habituated to the testing area both in groups of diminishing size and individually over an additional period of 5 days. Brown food bowls (RSPCA 18 cm Lettered Dog Bowls) containing crushed banana were also presented to pigs in both their home pen and in the middle of the testing area to create a positive association between the bowls and food.

### Food reward and reversal learning test

At 7 weeks of age half the pigs (n = 50) were selected pseudo-randomly (i.e. balanced for litter and sex) to perform the food reward test. This resulted in a sample of 26 socialised (11 male, 15 female) and 24 control (14 male, 10 female) individuals. Twelve food reward trials occurred across a 5-day period and involved training individuals to associate a randomly selected arm of the T-maze (Supplementary Fig. 1) with a food reward (i.e. the familiar food bowl baited with crushed banana). The location of the food reward remained consistent across all 12 trials. To minimise the effects of visual and olfactory cues, a non-baited bowl that had previously contained a food reward was located in the opposite arm of the T-maze (i.e. incorrect arm).

For each trial, pigs entered the maze via a centrally located gate and the time taken to find the baited bowl was recorded. Due to variation in food motivation, discovery of the reward was defined as the placing of the snout into the baited food bowl, rather than the consumption of food. The number of entries into each arm of the T-maze (counted when both front limbs crossed into the arm) was also recorded, in addition to the first arm visited.

Pigs were considered to have ‘successfully’ solved the T-maze if they located the food reward within 180 s of entering the maze without entering the incorrect arm. Individuals that entered the incorrect arm of the maze before locating the food were allowed to continue investigation but were recorded as having been ‘unsuccessful’. Pigs were able to make as many arm entries as required to find the food reward. Not all entries into the correct arm of the maze resulted in the location of the food reward. If the food reward had not been located within 180 s of entering the maze the individual was considered to have ‘failed’ the trial, given a maximum latency score of 180 s, and gently guided towards the baited bowl (i.e. a researcher entered the maze and directed the pig to the baited bowl). In order to ensure continued motivation, strawberry jam was added to the food bowls (in addition to the crushed banana) from the 6th trial onward after pigs had been habituated to the new reward in the home pen.

For progression to the reversal learning test pigs were required to perform a minimum of 5 of their final 6 food reward trials successfully, in addition to performing their first reversal learning trial unsuccessfully (i.e. pigs attempted to locate the food reward in its previously correct location). This resulted in a sample size of 13 individuals (6 socialised, 7 control) for the reversal learning test. Six reversal learning trials were performed in the same manner as the food reward trials although the baited bowl was relocated to the previously incorrect arm of the T-maze, while the non-baited bowl was placed in the previously correct location. Again, the time taken to discover the baited bowl, first arm entered, and the number of entries into each arm was recorded.

### Social preference test

The remaining 50 pigs (26 socialised, 24 control) that did not participate in the food reward/reversal learning tests were selected to perform a single social preference test in a modified version of the T-maze (Supplementary Fig. 2) that allowed for two pairs of unfamiliar stimulus pigs to be held in pens located on either side of the maze. Small and large stimulus pigs were selected from individuals that had previously performed the food reward trials based on their weight in relation to the average weight of the test pigs (Batch 1 = 19.89 ± SD 2.21 kg, Batch 2 = 17.25 ± SD 2.64 kg). On average, ‘small’ stimulus pigs were ~ 82% (SE ± 2%) of the test pig’s body weight, while ‘large’ stimuli pigs weighted ~ 123% (SE ± 3%). The same stimulus pigs were repeatedly used within a batch and were only replaced for tests involving familiar littermates. The side of the maze containing the small stimulus pigs was pseudo-randomised (i.e. balanced for litter and gender) in order to minimise the effect of any pre-existing side bias. During the trial, test pigs were separated from stimulus pigs by means of a division grid (Supplementary Fig. 3) that prevented physical interaction but allowed for visual and olfactory assessment. Both sides of the maze also contained equally baited food bowls (banana and strawberry jam) located near the division grid. Pigs were again introduced to the maze through a centrally located gate and allowed to move freely around the T-maze for 180 s. Latency to enter either side of the T-maze (again defined as the placement of both front limbs into the side), in addition to the proportion of time spent in each section of the maze, and the first side of the maze entered, was recorded. If a pig did not enter one of the sides it was given a maximum entry latency of 180 s for that side.

### Novel object tests

Due to the development of health concerns unrelated to testing one pig was removed from the experiment at this point and did not perform either of the novel object trials, resulting in a sample size of 99. Two novel object tests were performed between 8–9 weeks of age and a maximum of 36 h apart. Prior to the test an object with which the pigs had no previous experience was placed in the centre of the social preference T-maze (trial 1: traffic cone, trial 2: life ring). Pigs were then introduced to the maze and the time taken to make contact with the object was recorded. After initial contact pigs remained within the test area for 180 s and the percentage of time spent interacting with the novel object was recorded. If no contact was made within 300 s the test was ended and the individual was given a maximum latency of 300 s and an interaction time of 0 s. Neophobia scores were calculated for each test by subtracting an individual’s time spent interacting with the novel object from their latency to make contact with it (see^63^).

### Puzzle box tests

Each pig then performed two puzzle box trials in which the novel object from the previous test was replace by a novel 30 × 40 × 65 cm puzzle box (Supplementary Fig. 4). Puzzle box trials were performed on two consecutive days, a minimum of 48 h after the last novel object test. The puzzle box was constructed out of plywood and included a sliding clear Perspex door that pigs were able to move from side to side with their snout in order to gain access to a familiar food reward bowl containing crushed banana and strawberry jam. The sliding door was perforated with holes to allow olfactory cues to direct the pig’s attention towards the reward. Additionally, a light was installed inside the puzzle box in order to maximise the visibility of the food reward when the puzzle was shut.

The time taken for pigs to make contact with the puzzle box was recorded. If no contact was made within 180 s the test was ended and the individual was given the maximum latency score of 180 s. After first contact, pigs remained within the test area until either they successfully opened the puzzle box and obtained the food reward or until a futher 180 s had passed. The percentage of time a pig spent in contact with the puzzle box, as well as the time taken to solve it, was recorded. During the first test, if a pig did not solve the test within 3 min they were marked as unsuccessful and the box was opened for them in order to prevent negative associations being formed with the puzzle-box/test area.

### Statistical analysis

All data analysis was performed in the statistical package R version 3.3.1 (The R Foundation for Statistical Computing). All data are presented as means ± standard error of the mean.

i) Food Reward and Reversal Learning Test

Due to technical difficulties 8 of the 50 pigs selected for this test (5 socialised, 3 control) only participated in 11 of the 12 food reward trials. A further 10 trials from 9 pigs were ended before conclusion due to the performance of two escape attempts. Additionally, three pigs (1 socialisation, 2 control) were removed from the test and subsequent analysis due to incomplete habituation, as indicated by the performance of fear related behaviours during testing. This resulted in data for a total of 546 food reward trials (293 socialised, 253 control) from 47 pigs (25 socialised, 22 control). A total of 14 pigs (6 socialised, 8 control) met the learning criteria required for progression to the reversal learning trials. Of these, one control individuals performed the first reversal learning trial successfully (i.e. did not enter the now incorrect arm of the T-maze), suggesting that previous learning of the food reward location was not complete. This individual was therefore removed from the test and its subsequent analysis.

Results for the food reward T-maze test and the reversal learning T-maze test were analysed using separate sets of models. For both tests, the time taken to find the reward, the total number of entries into either side of the maze, the number of incorrect entries, and the number of correct entries that did or did not result in the individual locating the food reward were analysed as separate dependent outcomes using general linear mixed effects models (GLME’s). Trial number, treatment, sex, and all possible interactions were included in each of the models as fixed effects while batch, sow ID, and pig ID were included as appropriately nested random factors in order to account for the non-independence of individuals between trials. For food reward models, the location of the rewarded food bowl (i.e. right or left), and its interaction effects were also included as fixed effects. Due to a limited number of pigs progressing to the reversal learning trial, the previous location of the rewarded food bowl was included in these models as a fixed effect after all non-significant (*p* > 0.1) interaction effects were removed. Model residuals were checked for normality and data were transformed accordingly. Individual success (i.e. if an individual completed the trial with no incorrect entries) was examined in the same way using a generalised linear mixed effect model (GLMME) with a binomial distribution and a logit link function. The likelihood of an individual progressing to the reversal learning test was also examined using a GLMME containing treatment, sex, location of the rewarded food bowl, and all possible interactions as fixed effects. Pen ID and Batch were also included as nested random factors.

All models initially included all relevant variables and interaction effects. Non-significant interaction effects (*p* > 0.1) were removed from the model sequentially. All fixed factors were retained within the model, with the exception of food reward location, which was dropped if non-significant (*p* > 0.1). Test statistics were extracted from models using restricted maximum likelihood (REML) and a Wald’s Test.

ii) Social Preference Test

Analysis of individual behaviour during social preference testing included data gathered from all 50 participating piglets (26 socialised, 24 control). The percentage of time spent in each side of the T-Maze was examined using a GLME. The side of the T-maze occupied (i.e. the side containing the small stimulus pigs, hereafter referred to as the ‘small side’ vs the large stimulus pigs, hereafter referred to as the ‘large side’ vs the central area, hereafter referred to as the ‘middle’), an individual’s pre-weaning treatment (i.e. socialised vs control), sex, and the location of the small side (i.e. right or left) were all included in the model alongside their interactions as fixed effects while batch number and pen ID were also included in the models as appropriately nested random effects. The time taken for the piglets to enter into either side of the T-Maze and time taken to enter the small side of the T-maze was also explored using two GLMEs containing treatment, sex, the location of the small side and the interactions between them as fixed effects. Again, batch number and pen ID, were included as nested random effects in order to account for any underlying litter effects. Lastly, the likelihood of an individual to enter the side of the maze containing the pair of small stimulus pigs before entering the side of the large stimulus pigs was examined in the same way using a GLMME with a binomial distribution and a logit link function. Models initially contained all relevant fixed factors and interaction effects. Non-significant interactions (*p* > 0.1) were removed sequentially, while all fixed factors were retained. Models were examined by means of a Wald’s test and REML.

iii) Novel Object and Puzzle Box Test

As previously mentioned, one individual did not perform either of the novel object or puzzle box tests due to health concerns unrelated to testing. In addition to this, four other individuals did not complete the first novel object test due to escape attempts, while seven individuals did not complete the second novel object test. This resulted in a sample size of 94 individuals for the first test and 92 for the second test. Only three individuals did not receive a neophobia score for either test. Two individuals failed to make contact with the novel object during the second test and were subsequently given the maximum neophobic score of 300 for that test. When comparing the behaviour of individuals that completed both novel object trials (*n* = 90), neophobic score was found to be strongly correlated (Spearman’s Rank Correlation; *r*_*s*_ = 0.36, *p* < 0.01). Therefore, an average neophobia score was calculated for a total of 96 individuals. Average neophobia score was analysed using a GLME model containing treatment (socialised or control), sex, and all interaction effects as fixed factors. Batch and litter were also included as appropriately nested random factors. Initially all appropriate factors and interactions were included in the model. Non-significant interaction effects (*p* > 0.1) were removed from the model sequentially. All fixed factors were retained within the model. Once again, models were explored using REML and a Wald’s test.

During the first puzzle box trial two individuals failed to make contact with the puzzle and were given a maximum contact latency of 180 s. These incomplete trials were not included in models related to behaviours performed while interacting with the puzzle box. Additionally, nine individuals were removed from trial 1 and subsequent analysis due to escape attempts. Of these, four individuals were removed from trial 2 for the same reason. All individuals that performed trial 1 also performed trial 2. Trials containing individuals with no recorded neophobia score were also removed from analysis. This allowed latency to contact the puzzle box (hereafter referred to as contact latency) to be examined for 184 trials (trial 1 = 90, trial 2 = 94). The percentage of the trial that individuals spent in contact with the puzzle box (hereafter referred to as contact time), the latency to solve the puzzle box (hereafter referred to as solve latency), and the likelihood that an individual was able to solve the puzzle (1 or 0) were recorded for 182 trials (trial 1 = 88, trial 2 = 94). Individuals that did not solve the puzzle box within the initial 180 s after first contact were given the maximum latency to solve (180 s) and were recorded as non-successful.

GLME’s including treatment (socialised or control), experience with the puzzle (i.e. first or second attempt), sex, and average neophobia score as fixed effects were used to examine contact latency, contact time, and solve latency. Batch, litter, and focal piglet ID were also included in these models as appropriately nested random effects. Lastly, a GLMME with a binomial distribution and a logit link factor containing the aforementioned fixed and random effects was used to investigate the likelihood of successful access into the puzzle box.

## Results

### Food reward/reversal learning test

**Figure 1:**
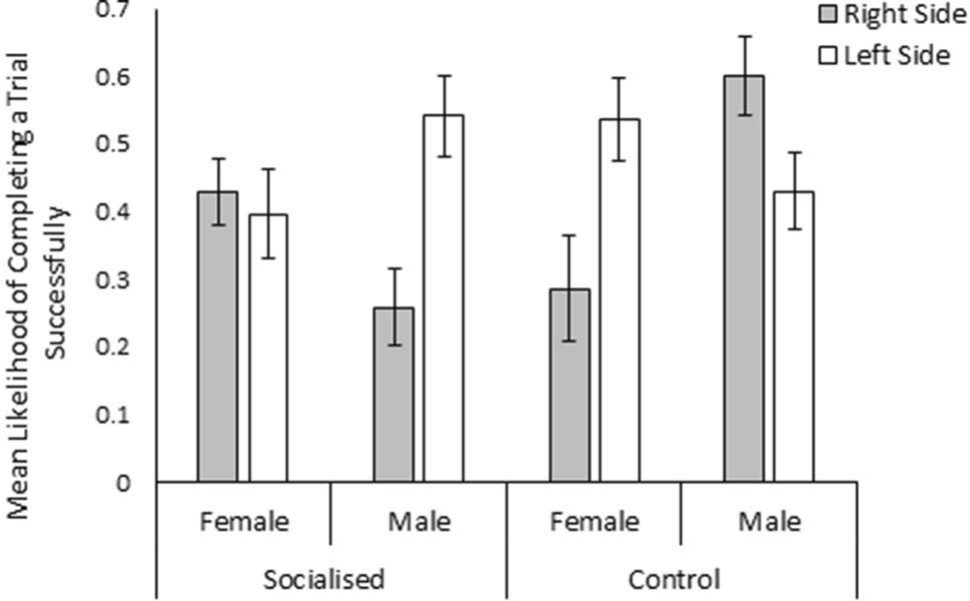
Mean likelihood of finding the food reward without entering into the incorrect arm of the food reward T-maze for socialised females, socialised males, control females, and control males when the food reward was located in the right side (grey bars) and left side (white bars) of the T-maze. Error bars represent the standard error of the mean.

**Figure 2:**
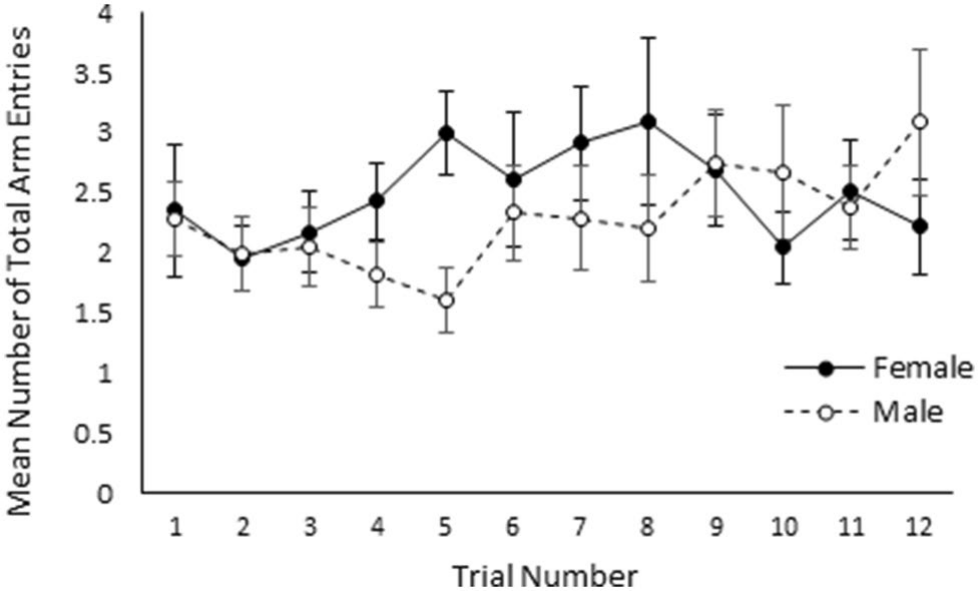
Mean number of total arm entries presented for females (black circles and solid black line) and males (white circles and dashed black line) across the 12 food reward trials. Error bars represent the standard error of the mean.

**Figure 3:**
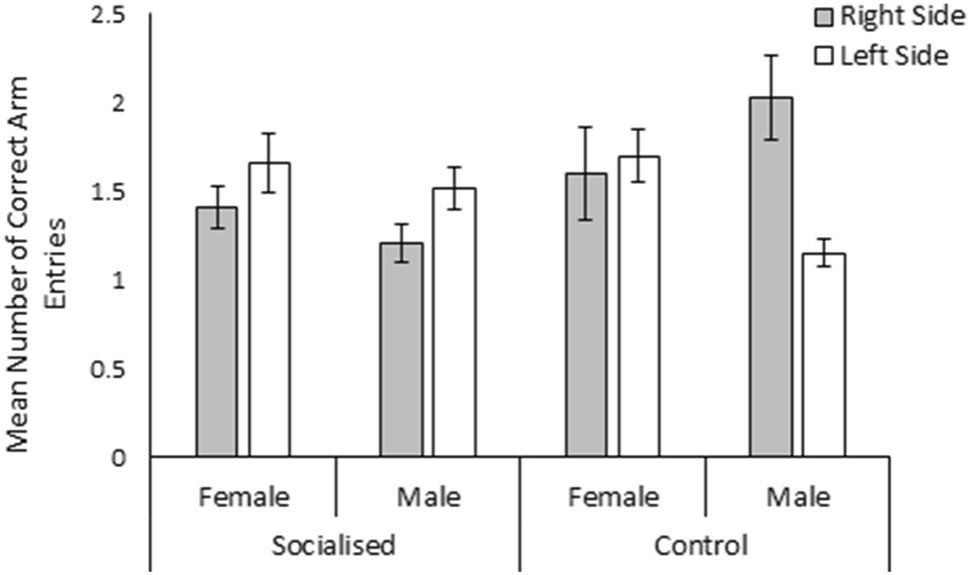
Mean number of correct arm entries made by socialised females, socialised males, control females, and control males during food reward trials when the food reward was located in the right side (grey bars) and left side (white bars) of the T-maze. Error bars represent the standard error of the mean.

The likelihood of an individual performing a food reward trial successfully was significantly affected by a three-way interaction between treatment, sex, and the location of the food reward (χ^2^_1_ = 3.96, *p* = 0.05, Fig. 1). Males from the socialised treatment and females from the control treatment were significantly more likely to complete a trial successfully when the food bowl was located in the left arm of T-maze than when it was located in the right arm (Mann Whitney U Test; *w* = 2679, *p* > 0.01 and *w* = 1510, *p* = 0.02 respectively). On the other hand, males from the control treatment were significantly more likely to successfully complete a trial when the food reward was located in the right arm of the T-Maze (Students T-Test; *w* = 2296, *p* = 0.04). The location of the food reward did not significantly influence the likelihood of socialised females performing the trial successfully (Students T-Test; *w* = 2947.5 *p* = 0.69). Additionally, the time taken to find the bowl containing the food reward, was not significantly influenced by sex, trial number, or treatment (*p* > 0.05). The total number of arm entries made was found to be significantly influenced by the interaction between sex and trial (χ^2^_11_ = 20.44, *p* = 0.04), although visualisation of the data revealed that males and females only differed in trial 5, where on average males made fewer total arm entries than females (male = 1.61 ± 0.27 entries, female = 3.00 ± 0.35 entries; Fig. 2). Additionally, the number of correct entries made was significantly affected by a three-way interaction between treatment, sex, and the side of the food reward (χ^2^_1_ = 4.67 ± 0.03). Visualisation of the data suggests that while no effect was observed in females, males from both the socialised and control treatment made more or less correct arm entries respectively when the baited food bowl was located in in the left arm of the T-maze (Fig. 3). The number of incorrect entries made was not significantly affected by sex, treatment, trial, or the location of the food reward bowl (*p* > 0.05). Furthermore, progression from the food reward test to the reversal learning test was not significantly influenced by treatment or sex (*p* > 0.05).

**Figure 4:**
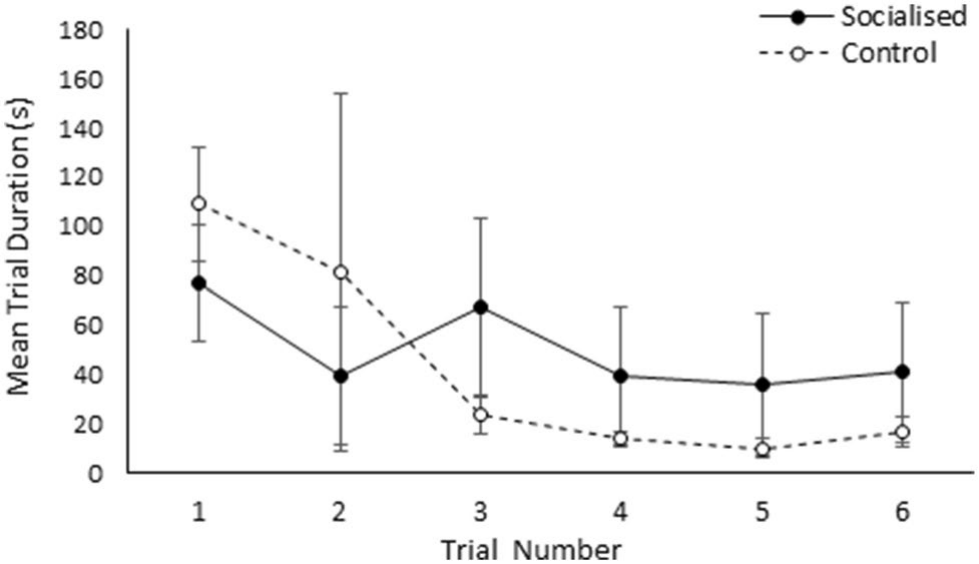
Mean latency (s) to find the food reward presented for socialised (black circles and solid black line) and control (white circles and dashed black line) individuals across the 6 reversal learning trials. Error bars represent the standard error of the mean.

The likelihood an individual performed a successful reversal learning trial was not significantly influenced by sex, trial number, treatment, or the previous location of the food reward (*p* > 0.05). However, latency to find the baited bowl did significantly change over the course of the 6 trials (χ^2^_5_ = 42.57, *p* < 0.01), with the extent of the change over time differing between treatments (χ^2^_5_ = 16.93, *p* < 0.01). Further investigation revealed that control individuals showed a large decrease in latency to find the baited bowl from the third trial onwards, while no change in the socialised treatment was observed over the course of the trials (Fig. 4).While the number of correct entries made was not significantly influenced by sex, trial number, treatment, or previous location of the food reward (*p* > 0.05), both the total number of arm entries and the number of incorrect entries were significantly influenced by trial (χ^2^_5_ = 23.11, *p* < 0.01 and χ^2^_5_ = 27.92, *p* < 0.01 respectively) and sex (χ^2^_1_ = 5.22, *p* = 0.02 and χ^2^_1_ = 3.94, *p* = 0.05 respectively). Both the total number of arm entries and the number of incorrect entries decreased over the course of the 6 trials, with the exception of trial 6 (Supplementary Material Fig. 5). Additionally, on average males performed fewer entries into the incorrect arm and subsequently performed fewer total arm entries than females (Supplementary Material Fig. 6).

### Social preference test

**Figure 5:**
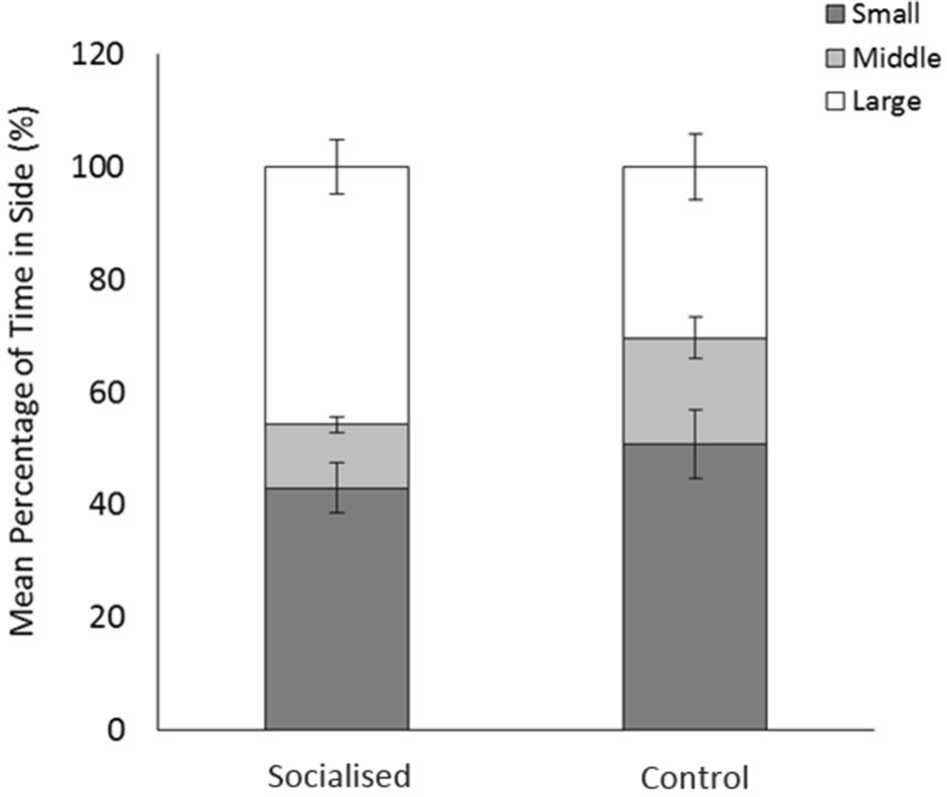
Mean percentage of time (max = 180 s) socialised and control individuals spent in the side of the T-maze containing either the small or large stimulus pigs during the Social Preference test. Percentage of time spent in the middle of the maze is also provided. Error bars represent the standard error of the mean.

The percentage of time piglets spent in each side of the maze was significantly affected by the size of the stimulus pigs (χ^2^_1_ = 52.07, *p* < 0.01), with individuals on average spending longer in the small side than the large side (Tukey’s Honest Significant Differences Test; *z* = 3.11, *p* < 0.01), or the middle (*z* = 4.86, *p* < 0.01) of the maze (small side: 47 ± 4%; middle: 15 ± 2%; large size: 38 ± 4%). There was also a significant interaction effect of treatment and stimulus pig size on time spent in each side of the maze (χ^2^_2_ = 8.43, *p* = 0.01), with socialised individuals spending longer in the large side of the T-maze than controls (and subsequently less time in the middle of the T-maze; Fig. 5).

There was no significant effect of treatment or sex on an individual’s likelihood of entering the small side first, although latency to enter the small side for the first time was significantly influenced by the three-way interaction effect of treatment, sex, and the location of the small side (χ^2^_1_ = 6.62, *p* = 0.01; Supplementary Material Fig. 7). When the small stimulus pigs were located on the left, socialised females and control males were quicker to enter the small side than when the small stimulus pigs were located on the right. On the other hand, socialised males and control females appeared quicker to enter the small side when it was located on the right compared to the left. Upon further investigation, however, these observations were found to be non-significant (Students T-Tests and Mann Whitney U Tests; all *p* > 0.1).

**Figure 6:**
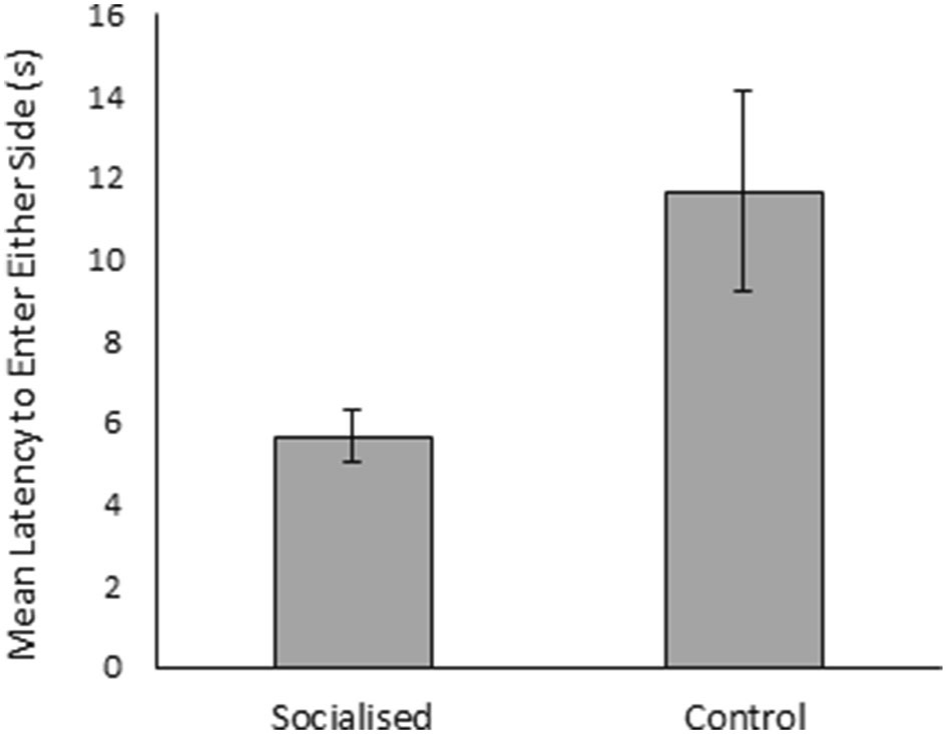
Mean latency (s) of socialised and control individuals to enter either side of the of social preference test. Error bars represent the standard error of the mean.

Latency to enter into either side of the T-maze was significantly influenced by treatment (χ^2^_1_ = 12.16, *p* < 0.01; Fig. 6), the two-way interaction between sex and small side location (χ^2^_1_ = 9.16, *p* < 0.01), and the three-way interaction between treatment, sex, and small side location (χ^2^_1_ = 4.39, *p* = 0.04; Supplementary Material Fig. 8). Overall, control pigs took significantly longer to leave the middle of the T-maze than socialised pigs. The side of the maze in which the small stimulus pigs were located did not significantly affect the latency of socialised males, socialised females, or control males to enter either side of the maze (Students T-Tests and Mann Whitney U Tests; all *p* > 0.2). Females from the control treatment appeared quicker to leave the middle of the maze when the small stimulus pigs were located on the right, however this was not significant (Mann Whitney U Test; W = 20.5, *p* = 0.75).

### Novel object test and puzzle box test

**Figure 7:**
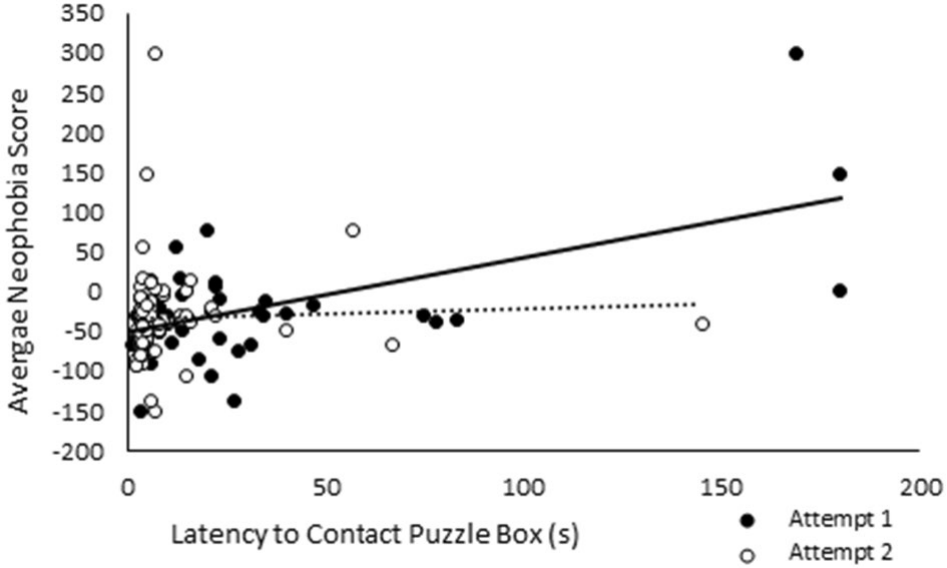
The relationship between average neophobia scores and latency to contact the puzzle box (s) presented for both attempts (attempt 1—black circles and solid black trendline, attempt 2—white circles and dashed black trendline).

Average neophobia score was not significantly influenced by treatment or sex. As predicted, latency to contact the puzzle box was significantly influenced by both average neophobia score (χ^2^_1_ = 13.24, *p* < 0.01) and attempt number (χ^2^_1_ = 13.14, *p* < 0.01). A significant two-way interaction revealed that the extent to which average neophobia score influenced latency to contact the puzzle box was significantly influenced by attempt number (χ^2^_1_ = 11.89, *p* < 0.01; Fig. 7); latency to contact the puzzle box was significantly correlated with average neophobia score during the pigs’ first attempt to solve the puzzle box (Spearman’s Rank Correlation; r_s_ = 0.34, D.F. = 89, *p* < 0.01) but not their second attempt (Spearman’s Rank Correlation; r_s_ = 0.19, D.F. = 93, *p* = 0.07). Latency to contact the puzzle box was also significantly influenced by a two-way interaction between sex and average neophobia score (χ^2^_1_ = 4.07, *p* = 0.04; Supplementary Material Fig. 9) and a four-way interaction between treatment, sex, attempt number, and average neophobia score (χ^2^_1_ = 5.31, *p* = 0.02), although no obvious patterns could be discerned. Across all individuals, pig spent longer interacting with the puzzle box on their second attempt than their first (χ^2^_1_ = 5.85, *p* = 0.02) and were, on average, quicker to solve the puzzle box during their second attempt (χ^2^_1_ = 6.78, *p* < 0.01).

During the first trial, 51 of the 88 pigs that made contact with the puzzle box were able to solve it within 180 s. During the second trial 54 of the 94 pigs that made contact with the puzzle box were able to solve it within 180 s. 82% of individuals that succeeded in opening the puzzle box on their first attempt were also successful during their second attempt. However, neither treatment, sex, neophobia score, nor trial number influenced an individual’s likelihood of solving the puzzle.

## Discussion

We hypothesised that over both the 12 trials of the food reward test and the 6 trials of the reversal learning test, individuals would improve the speed with which they were able to locate the baited reward bowl, while decreasing the number of entries made into the incorrect arm of the maze. Furthermore, given previous evidence suggesting that early life social environment can influence brain development^30–32^, and that environmental enrichment can lead to improved working memory in pigs^28^, we predicted that socialisation would influence the rate at which individuals showed this improvement, with socialised individuals improving more rapidly than control individuals. Despite this, trial number did not appear to influence behaviour during the food reward test in any meaningful way. However, over the course of the six reversal learning trials the average number of entries into the unrewarded arm of the T-maze (and subsequently the total number of arm entries) did decrease as trial number progressed, indicating a possible reliance on reference memory. Furthermore, latency to find the food reward during the reversal learning trials did appear to decrease between trials 2 and 3 in control individuals, although contrary to our predictions no obvious change was observed in individuals from the socialised treatment. This result should be interpreted with caution however, as only individuals that reached the learning criteria in the food reward test participated in these trials, resulting in a comparatively small sample size (n = 13). Additionally, the likelihood of an individual entering into the side of the T-maze containing the food reward before entering the incorrect side did not change over the course of either test, suggesting that pigs may not have remembered the location of the food bowl from one trial to the next.

These findings somewhat contrast those of previous studies, which have reported clear evidence of reference memory use in pigs during spatial discrimination tasks [^8,27,28,64,65^, but see^38^]. We can think of three reasons as to why our study showed little evidence for reference memory use, especially during the food reward task, despite the findings of these previous studies. The first is that the young age of the pigs during this test (7 weeks old) may have limited their ability to recall and act upon prior experience. Under natural conditions, piglets do not begin weaning until between 8 and 15 weeks of age^66^. The continuous presence and care of the sow prior to this may limit the need for piglets to develop certain cognitive abilities (such as spatial memory) during this period. Secondly, given the reduced spatial complexity of a T-maze, the food reward and reversal learning tasks presented in this experience were likely to have been less cognitively demanding than those previously used to explore working and reference memory (i.e. spatial holeboard task^28,64^). Held et al.^6^ argued that memory-based tasks might need to be more complex than remembering one food location if looking for an effect of early-life stressors on cognition. It is possible that the limited number of potential food locations, in addition to the lack of a negative reinforcement during incorrect choices, may have reduced the cost of simply re-exploring the T-maze during every trial, making reliance upon reference memory redundant. Given the age of the pigs and the potential impact negative experience may have had on both welfare and motivation to enter the T-maze, the implementation of a negative reinforcer was not suitable for this study, although it may lead to improved learning in older individuals and should be considered for further studies. Lastly, a larger number of trials may have been needed to detect an improvement in reference memory, especially for individuals that were unable to achieve the learning criteria required for progression to the reversal learning test. This final suggestion seems unlikely however, as improvements to spatial memory have been observed in pigs over the course of considerably fewer trials^8,65^.

During the reversal learning test, females were found to make significantly more incorrect arm entries (and subsequently more total arm entries) than their male counterparts. Again, this result should be interpreted with caution due to the small number of individuals progressing to the reversal learning test, however, sexual dimorphism in cognitive abilities has been reported in a variety of species^67–70^. Interestingly, previous studies considering the effect of sex on spatial cognition in the domestic pig have been contradictory^8,27,71,72^. Sex differences in cognitive ability may be explained by differences in the environmental demands males and females experience, such as social life complexity or home range size^72–74^. For example, male and female wild boar experience very different adult social environments, with females forming groups of closely related individuals^75,76^ and males typically dispersing from their maternal group at ~ 16 months of age to become solitary^75,77,78^. During the mating season, males will compete vigorously with each other for access to these groups and subsequently chase away any males over a year old that have remained with the group^77^. Therefore, in order to maximise their breeding potential, solitary males typically hold a much larger home range than groups of females^77,79^. This increased home range, in addition to the absence of potentially informed conspecifics from whom information can be exploited^46,47^ may explain why males in our study made fewer mistakes than their female counterparts during the reversal learning test.

Although it was hypothesised that socialised individuals would show greater reversal learning due to their pre-weaning experiences, this was not the case. Control individuals were observed to be quicker in adapting to a change in food location than their socialised counterparts. This is in line with the findings of Mendl et al*.*^38^, who observed that pigs raised in an enriched environment were less able to supress their previously learned response to a T-Maze than individuals from a barren environment. Furthermore, De Jong et al*.*^27^ observed that while pigs raised in a barren environment were initially more fearful of a novel situation, they were quicker to habituate to this novelty than individuals raised in an enriched environment. It is therefore possible that the absence of non-littermates may have resulted in a lower-quality environment (such as those experienced in previous studies) for the control individuals, subsequently resulting in an improved ability to display flexible learning.

An alternative explanation for this improved behavioural flexibility in control individuals may have been an increased motivation to explore the T-maze, as experience of a barren early life environment has been suggested to increase boldness and exploratory behaviour in domestic pigs (^38,60^ but see^65,80^). However, little evidence was found in this study to support this theory as control individuals did not differ from their socialised counterparts in the number of arm entries made throughout the reversal learning test. Furthermore, treatment did not affect neophobia score as calculated during the novel object tests, suggesting socialised and control individuals did not differ in their motivation to perform exploratory behaviours. Contrary to predictions, socialised individuals showed no improvement over the course of the reversal learning test, suggesting they were less able to deviate from a previously learned behaviour than their control counterparts.

Lastly, the location of the food reward bowl (i.e. right or left) appeared to significantly interact with other factors explored within this study. For example, a three-way interaction between treatment, sex, and the location of the food reward was observed to influence both the likelihood of success in a trial and the number of correct arm entries made during the food reward tests. While no obvious pattern could be discerned, we can think of two reasons why the arm in which the food reward was located may have influenced results. Firstly, individual differences in lateralisation caused by sex and early life social experience may have led to side preferences during the food reward test^81^. Alternatively, pigs may have developed a bias side preference due to the location of their home pen relative to that of the T-maze. In order to reach the T-maze, some individuals may have had to turn repeatedly in one direction, while others may have experienced a more balanced number of right and left turns.

As predicted, during the social preference test pigs spent significantly different amounts of time in the three sections of the T-maze, showing an overall preference for the side containing the small stimulus pigs. However, socialised and control individuals were found to significantly differ in the percentage of time spent in each side of the maze. Socialised individuals on average spent nearly half (46%) of the trial in the large side of the T-maze, while control individuals spent a larger percentage of the trial in the middle of the T-maze than their socialised counterparts. It was hypothesised that socialised pigs would be better able to distinguish differences in the RHP of the stimulus pigs, and would therefore show a greater preference for eating at the food bowl nearest to the small stimulus pigs, over who they would be better able to assert their dominance. However, although socialised pigs were quicker than controls to leave the middle of the T-maze, indicating a more rapid assessment, they spent significantly more time in the side of the large stimulus pig than their control counterparts. We suggest that socialised individuals may have been less fearful in the presence of unfamiliar pigs, having already experienced novel social interactions pre-weaning. This would explain why they were quicker to enter into either side of the maze, and why they appeared less reluctant to feed from the food bowl located in the same side of the T-maze as the large stimulus pigs. Previous studies have demonstrated improved social behaviour in adult pigs experiencing early life socialisation during the pre-weaning period, such as reduced aggression^17,18,21–24^, while studies exploring the effects of early life physical enrichment on social interactions observed that pigs from an enriched environment directed less manipulative behaviour (such as nosing and tail-biting) towards pen-mates than those experiencing a barren pre-weaning environment^60,82,83^. Furthermore, a study exploring the effect of early life social environment of mice found that individuals raised in a communal litter displayed a higher propensity to interact socially and were quicker to achieve a defined social role than those raised within standard nests^30^.

Alternatively, control pigs may have been better able to assess the RHP of the stimulus pigs than those experiencing socialisation. A recent study suggested that control individuals performed a form of opponent-only assessment, engaging less with larger opponents, regardless of their own RHP^84^. In contrast, pigs experiencing socialisation pre-weaning were observed to perform a novel form of assessment in which they gather information regarding both the RHP of themselves and of their opponents^84,85^. Socialised individuals were more prepared to further escalate contests when the RHP difference between themselves and their opponent was large and in the opponents’ favour, which may explain the observed preference of socialised pigs for the large side of the T-maze.

Despite this, treatment did not affect the likelihood of an individual entering the side of the T-Maze containing the small individuals first, although again a three-way interaction effect between treatment, sex, and the location of the small side suggested the potential development of side biases. It is possible that test individuals were unable to gather accurate information regarding the stimulus pigs RHP, such as their relative size, from the middle of the maze, despite the short distance. Pigs perform parallel walking and adopt a shoulder to shoulder position before performing escalated contest behaviours^50,78,86^, which may be necessary for assessment. As such pigs may have entered into a side at random before beginning their assessment.

While individual neophobia score was found to be correlated between the two novel object trials, it was not significantly influenced by sex or treatment. This was surprising as previous studies have reported an increased motivation for exploration in pigs experiencing a barren pre-weaning environment^38,58–60^. It is probable that while the absence of non-littermates limited the range of potential play partners pigs had access to, neither socialised nor control individuals differed in their pre- or post-weaning experience of abiotic factors, such as space per pig (i.e. stocking density) or substrate availability.

It was predicted that socialised pigs would be quicker to solve the puzzle box than their control counterpart once neophobia (and subsequently their motivation to perform exploratory behaviours) was accounted for. However, we found no evidence to suggest that socialised or control pigs differed in their motivation to investigate novel objects or their innovation with regards to a puzzle box task. Latency of individuals to approach the puzzle box was influenced by their neophobia score, with the extent of this effect being influenced by both trial and sex. As expected, pigs were less fearful of the puzzle box during their second exposure and were significantly quicker to make contact. Furthermore, the average time taken to solve the puzzle decreased between trials, although the likelihood of success did not differ. This suggests that while pigs that solved the puzzle on their first attempt were quicker to solve it during their second, pigs that did not solve it remained unable to do so even after having the solution shown to them by researchers.

It should be noted that due to the large number of statistical tests used in the analysis, the occurrence of Type 1 error was possible, and these results should therefore be interpreted with some caution [see^87^]. However, previous studies exploring the influence of early life social environment on pig behaviour have shown such effects can often be very subtle and may be missed when research is focussed on overall outcomes, rather than individual components of behaviour^84^. Additionally, Nakagawa^88^ has reported that behavioural research is more likely to suffer from Type 2 errors than other fields, due to the practical and ethical limitations of testing large numbers of animals. Therefore, the use of significance correction techniques (such as the Bonferroni correction) to account for the possibility of Type 1 errors would likely have further increased the risk of reporting Type 2 errors to unacceptable levels.

## Conclusion

In conclusion we found no evidence to suggest that socialised individuals relied on reference memory over the course of the food reward tests. However, individuals that reached the learning criteria of the reversal learning test did reduce their total number of entries and the number of incorrect entries made over the course of 6 trials, suggesting some evidence of reference memory use. Furthermore, control individuals were observed to be better able to alter their behaviour when the location of the food reward was switched than socialised individuals, as indicated by a reduction in latency to find the food reward between trials 2 and 3. During the social preference test individuals were observed to spend longer in the side of the maze containing the small stimulus pigs than in the side of the maze containing the large stimulus pigs, or the middle. Socialised individuals were quicker than controls to leave the middle of the maze and were found to spend longer in the side of the maze containing the large stimulus pigs, suggesting an increase in boldness. This adds further support to previous studies suggesting socialised pigs may prefer to perform escalated contest behaviour when their opponent displays a higher RHP than their own. Neither neophobia score nor ability to innovate was influenced by treatment or sex. Overall, these data provide some evidence to suggest that socialisation influences social cognitive development as revealed by the social preference test results. However, other aspects of cognitive development were not affected, suggesting that other mechanisms to explain the changes in social behaviour observed following early-life socialisation should be explored.

## Supplementary information

Supplementary Information

## Data Availability

Data is available upon request.

## References

[1] Shettleworth SJ (2001). Animal cognition and animal behaviour. Anim. Behav..

[2] Pearce JM (2008). Animal Learning and Cognition: An Introduction.

[3] Mendl M, Held S, Byrne RW (2010). Pig cognition. Curr. Biol..

[4] Gieling ET, Nordquist RE, van der Staay FJ (2011). Assessing learning and memory in pigs. Anim. Cognit..

[5] Marino, L. & Colvin, C. M. Thinking pigs: a comparative review of cognition, emotion, and personality in *Sus domesticus*. *Int. J. Comp. Psychol*. **28**, 23859 (2015).

[6] Held S, Mendl M, Laughlin K, Byrne RW (2002). Cognition studies with pigs: livestock cognition and its implication for production. J. Anim. Sci..

[7] Wechsler B, Lea SE (2007). Adaptation by learning: its significance for farm animal husbandry. Appl. Anim. Behav. Sci..

[8] Sneddon IA, Beattie VE, Dunne L, Neil W (2000). The effect of environmental enrichment on learning in pigs. Anim. Welf..

[9] Tanida H, Nagano Y (1998). The ability of miniature pigs to discriminate between a stranger and their familiar handler. Appl. Anim. Behav. Sci..

[10] Boissy A (2007). Assessment of positive emotions in animals to improve their welfare. Physiol. Behav..

[11] Broom DM (2010). Cognitive ability and awareness in domestic animals and decisions about obligations to animals. Anim. Behav. Sci..

[12] Newberry RC, Wood-Gush DGM (1986). Social relationships of piglets in a semi-natural environment. Anim. Behav..

[13] Petersen HV, Vestergaard K, Jensen P (1989). Integration of piglets into social groups of free-ranging domestic pigs. Appl. Anim. Behav. Sci..

[14] D'Eath RB, Lawrence AB (2004). Early life predictors of the development of aggressive behaviour in the domestic pig. Anim. Behav..

[15] Donaldson TM, Newberry RC, Špinka M, Cloutier S (2002). Effects of early play experience on play behaviour of piglets after weaning. Appl. Anim. Behav. Sci..

[16] Meese GB, Ewbank R (1973). The establishment and nature of the dominance hierarchy in the domesticated pig. Anim. Behav..

[17] D’Eath RB (2005). Socialising piglets before weaning improves social hierarchy formation when pigs are mixed post-weaning. Appl. Anim. Behav. Sci..

[18] Verdon M, Morrison RS, Hemsworth PH (2016). Rearing piglets in multi-litter group lactation systems: effects on piglet aggression and injuries post-weaning. Appl. Anim. Behav. Sci..

[19] Ewbank R, Bryant MJ (1972). Aggressive behaviour amongst groups of domesticated pigs kept at various stocking rates. Anim. Behav..

[20] Signoret, J. P., Baldwin, B. A., Fraser, D. & Hafez, E. S. E. The behaviour of swine. In *Behaviour of Domestic Animals* 3rd edn. (ed. Hafez, E. S. E.) 295–329 (Baillière Tindall, 1975)

[21] Wattanaku W, Stewart AH, Edwards SA, English PR (1997). Effects of grouping piglets and changing sow location on suckling behaviour and performance. Appl. Anim. Behav. Sci..

[22] Parratt CA (2006). The fighting behaviour of piglets mixed before and after weaning in the presence or absence of a sow. Appl. Anim. Behav. Sci..

[23] Kutzer T, Bünger B, Kjaer JB, Schrader L (2009). Effects of early contact between non-littermate piglets and of the complexity of farrowing conditions on social behaviour and weight gain. Appl. Anim. Behav. Sci..

[24] Camerlink I, Farish M, D’Eath R, Arnott G, Turner S (2018). Long term benefits on social behaviour after early life socialization of piglets. Animals.

[25] Weller JE, Camerlink I, Turner SP, Farish M, Arnott G (2019). Socialisation and its effect on play behaviour and aggression in the domestic pig (*Sus scrofa*). Sci. Rep..

[26] Salazar LC (2018). Early socialisation as a strategy to increase piglets’ social skills in intensive farming conditions. Appl. Anim. Behav. Sci..

[27] De Jong IC (2000). Effects of environmental enrichment on behavioral responses to novelty, learning, and memory, and the circadian rhythm in cortisol in growing pigs. Physiol. Behav..

[28] Bolhuis JE (2013). Working and reference memory of pigs (*Sus scrofa domesticus*) in a holeboard spatial discrimination task: the influence of environmental enrichment. Anim. Cogn..

[29] Olton DS, Becker JT, Handelmann GE (1979). Hippocampus, space, and memory. Behav. Brain Sci..

[30] Branchi I, D’Andrea I, Fiore M, Di Fausto V, Aloe L, Alleva E (2006). Early social enrichment shapes social behavior and nerve growth factor and brain-derived neurotrophic factor levels in the adult mouse brain. Biol. Psychiatry.

[31] Trokovic N, Gonda A, Herczeg G, Laurila A, Merilä J (2011). Brain plasticity over the metamorphic boundary: carry-over effects of larval environment on froglet brain development. J. Evol. Biol..

[32] Fischer S, Bessert-Nettelbeck M, Kotrschal A, Taborsky B (2015). Rearing-group size determines social competence and brain structure in a cooperatively breeding cichlid. Am. Nat..

[33] Humphrey, N. The social function of intellect. In *Growing Points in Ethology* (eds. Bateson, P. P. G. & Hind, R. A.) 303–317 (Cambridge University Press, 1976).

[34] Ashton BJ, Thronton A, Ridley AR (2018). An intraspecific appraisal of the social intelligence hypothesis. Philos. Trans. R. Soc. B Biol. Sci..

[35] Bond AB, Kamil AC, Balda RP (2003). Social complexity and transitive inference in corvids. Anim. Behav..

[36] Emery NJ, Seed AM, Von Bayern AM, Clayton NS (2007). Cognitive adaptions of social bonding in birds. Philos. Trans. R. Soc. B. Biol. Sci..

[37] Lukas D, Clutton-Brock T (2018). Social complexity and kinship in animal societies. Ecol. Lett..

[38] Mendl M, Erhard HW, Haskell M, Wemelsfelder F, Lawrence AB (1997). Experience in substrate-enriched and substrate-impoverished environments affects behaviour of pigs in a T-maze task. Behaviour.

[39] Bolhuis JE, Schouten WG, de Leeuw JA, Schrama JW, Wiegant VM (2004). Individual coping characteristics, rearing conditions and behavioural flexibility in pigs. Behav. Brain Res..

[40] Cox LN, Cooper JJ (2001). Observations on the pre- and post-weaning behaviour of piglets reared in commercial indoor and outdoor environments. Anim. Sci..

[41] Gamboa GJ, Reeve HK, Holmes WG (1991). Conceptual issues and methodology in Kin-recognition research: a critical discussion. Ethology.

[42] Seyfarth, R. M. & Cheney, D. L. How monkeys see the world: a review of recent research on East African vervet monkeys. In *Primate Communication* (ed. Snowdon, C. *et al*.) 239–252 (Cambridge University Press, 1981).

[43] Cheney DL, Seyfarth RM (1980). Vocal recognition in free-ranging vervet monkeys. Anim. Behav..

[44] Cheney DL, Seyfarth RM (1999). Recognition of other individuals’ social relationships by female baboons. Anim. Behav..

[45] Premack D, Woodruff G (1978). Does the chimpanzee have a theory of mind?. Behav. Brain Sci..

[46] Held S, Mendl M, Devereux C, Byrne RW (2000). Social tactics of pigs in a competitive foraging task: the ‘informed forager’ paradigm. Anim. Behav..

[47] Held S, Mendl M, Devereux C, Byrne RW (2002). Foraging pigs alter their behaviour in response to exploitation. Anim. Behav..

[48] Parker GA (1974). Assessment strategy and the evolution of fighting behaviour. J. Theor. Biol..

[49] Maynard Smith J (1974). The theory of game and the evolution of animal conflicts. J Theor Biol..

[50] Camerlink I, Turner SP, Farish M, Arnott G (2015). Aggressiveness as a component of fighting ability in pigs using a game-theoretical framework. Anim. Behav..

[51] Weller JE, Turner SP, Farish M, Camerlink I, Arnott G (2020). The Association between play fighting and information gathering during subsequent contests. Sci. Rep..

[52] Arnott G, Elwood RW (2009). Assessment of fighting ability in animal contests. Anim. Behav..

[53] Fawcett TW, Mowles SL (2013). Assessments of fighting ability need not be cognitively complex. Anim. Behav..

[54] Elwood RW, Arnott G (2013). Assessments in contests are frequently assumed to be complex when simple explanations will suffice. Anim. Behav..

[55] Benson-Amram S, Holekamp KE (2012). Innovative problem solving by wild spotted hyenas. Proc. R. Soc. B.

[56] Manrique HM, Völter CJ, Call J (2013). Repeated innovation in great apes. Anim. Behav..

[57] Sol D, Duncan RP, Blackburn TM, Cassey P, Lefebvre L (2005). Big brains, enhanced cognition, and response of birds to novel environments. Proc. Natl. Acad. Sci. U. S. A..

[58] Stolba A, Wood-Gush DGM (1980). Arousal and exploration in growing pigs in different environments (abstr). Appl. Anim. Ethol..

[59] Pearce GP, Paterson AM (1993). The effect of space restriction and provision of toys during rearing on the behaviour, productivity and physiology of male pigs. Appl. Anim. Behav. Sci..

[60] De Jong IC (1998). Effects of strawbedding on physiological responses to stressors and behavior in growing pigs. Physiol. & Behav..

[61] Overington SE, Cauchard L, Côté KA, Lefebvre L (2011). Innovative foraging behaviour in birds: what characterizes an innovator?. Behav. Process..

[62] Auersperg AM, Von Bayern AM, Gajdon GK, Huber L, Kacelnik A (2011). Flexibility in problem solving and tool use of kea and New Caledonian crows in a multi access box paradigm. PLoS ONE.

[63] Kareklas K, Arnott G, Elwood RW, Holland RA (2016). Plasticity varies with boldness in a weakly-electric fish. Front. Zool..

[64] Arts JW, van der Staay FJ, Ekkel ED (2009). Working and reference memory of pigs in the spatial holeboard discrimination task. Behav. Brain. Res..

[65] Jansen J, Bolhuis JE, Schouten WG, Spruijt BM, Wiegant VM (2009). Spatial learning in pigs: effects of environmental enrichment and individual characteristics on behaviour and performance. Anim. Cogn..

[66] Newberry RC, Wood-Gush DG (1985). The suckling behaviour of domestic pigs in a semi-natural environment. Behaviour.

[67] Gaulin SJ, FitzGerald RW (1986). Sex differences in spatial ability: an evolutionary hypothesis and test. Am. Nat..

[68] Seymoure P, Dou H, Juraska JM (1996). Sex differences in radial maze performance: influence of rearing environment and room cues. Psychobiology.

[69] Andreano JM, Cahill L (2009). Sex influences on the neurobiology of learning and memory. Learn. Memory.

[70] Lucon-Xiccato T, Bisazza A (2014). Discrimination reversal learning reveals greater female behavioural flexibility in guppies. Biol. Lett..

[71] Siegford JM, Rucker G, Zanella AJ (2008). Effects of pre-weaning exposure to a maze on stress responses in pigs at weaning and on subsequent performance in spatial and fear-related tests. Appl. Anim. Behav. Sci..

[72] Roelofs S, Nordquist RE, van der Staay FJ (2017). Female and male pigs’ performance in a spatial holeboard and judgment bias task. Appl. Anim. Behav. Sci..

[73] Healy SD, Bacon IE, Haggis O, Harris AP, Kelley LA (2009). Explanations for variation in cognitive ability: behavioural ecology meets comparative cognition. Behav. Process..

[74] Lucon-Xiccato T, Dadda M, Bisazza A (2016). Sex differences in discrimination of shoal size in the guppy (*Poecilia reticulata*). Ethology.

[75] Gabor TM, Hellgren EC, Van Den Bussche RA, Silvy NJ (1999). Demography, sociospatial behaviour and genetics of feral pigs (*Sus scrofa*) in a semi-arid environment. J. Zool..

[76] Kaminski G, Brandt S, Baubet E, Baudoin C (2005). Life-history patterns in female wild boars (*Sus scrofa*): mother–daughter postweaning associations. Can. J. Zool..

[77] Tisdell, C. A. *Wild Pigs: Environmental Pest or Economic Resource?* (Pergamon Press Pty Ltd, 1982).

[78] Barrette C (1986). Fighting behavior of wild *Sus scrofa*. J. Mammal..

[79] Baber DW, Coblentz BE (1986). Density, home range, habitat use, and reproduction in feral pigs on Santa Catalina Island. J. Mammal..

[80] Wemelsfelder F, Haskell M, Mendl MT, Calvert S, Lawrence AB (2000). Diversity of behaviour during novel object tests is reduced in pigs housed in substrate-impoverished conditions. Anim. Behav..

[81] Vallortigara G, Roger L (2005). Survival with an asymmetrical brain: advantages and disadvantages of cerebral lateralization. Behav. Brain. Sci..

[82] Schouten, W. G. P. Rearing conditions and behaviour in pigs. Dissertation, Wageningen Agriculutal (1986).

[83] Beattie VE, Walker N, Sneddon IA (1996). An investigation of the effect of environmental enrichment and space allowance on the behaviour and production of growing pigs. Appl. Anim. Behav. Sci..

[84] Weller JE, Camerlink I, Turner SP, Farish M, Arnott G (2019). Playful pigs: early life play-fighting experience influences later life contest dynamics. Anim. Behav..

[85] Camerlink I, Turner SP, Farish M, Arnott G (2019). Advantages of social skills for contest resolution. Roy. Soc. Open Sci..

[86] Camerlink I, Arnott G, Farish M, Turner SP (2016). Complex contests and the influence of aggressiveness in pigs. Anim. Behav..

[87] Luke SG (2017). Evaluating significance in linear mixed-effects models in R. Behav. Res. Methods.

[88] Nakagawa S (2004). A farewell to Bonferroni: the problems of low statistical power and publication bias. Behav. Ecol..

