# Supplementary material for: The influence of early life socialisation on cognition in the domestic pig (Sus scrofa domestica)
Source: Sci Rep. 2020 Nov 5;10:19077. doi: 10.1038/s41598-020-76110-5 (PMC7644636; doi:10.1038/s41598-020-76110-5)
Supplement: Supplementary file 1 — Supplementary Information [file 41598_2020_76110_MOESM1_ESM.pdf]

The influence of early life socialisation on cognition in the domestic pig (*Sus scrofa domestica*)

Jennifer E. Weller[1,2*], Simon P. Turner[3], Agnieszka Futro[3], Jo Donbavand[3], Mark Brims[3], Gareth Arnott[1]

[1)] *Institute for Global Food Security, School of Biological Sciences, Queens University Belfast, Belfast. U.K*

[2)] *Department of Comparative Pathobiology, Purdue University, West Lafayette, Indiana, USA*

[3)] *Animal Behaviour & Welfare, Scotland's Rural College (SRUC), Edinburgh, U.K*

[*] Corresponding Author: jeweller@purdue.edu

Supplementary Material

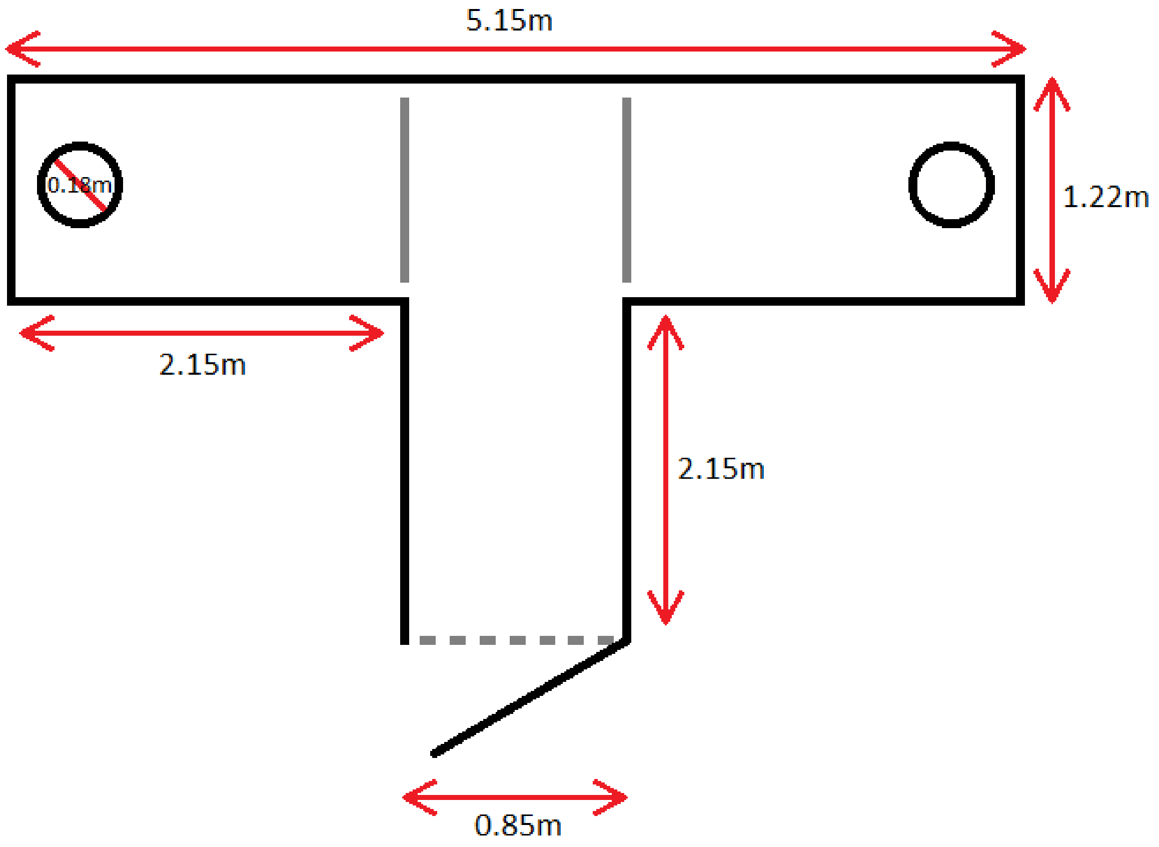

Supplementary Figure 1 – The T-maze used for Food Reward and Reversal Learning trials. Solid Grey lines indicate division of branches from the central branch and were not visible to the test individual during the trial. The dashed grey line indicates the location of the gate when in the closed position. Rewarded food bowls (Brown Lettered Dog Bowl – 18cm Diameter, RSPCA) contained either just crushed banana or crushed banana and strawberry jam.

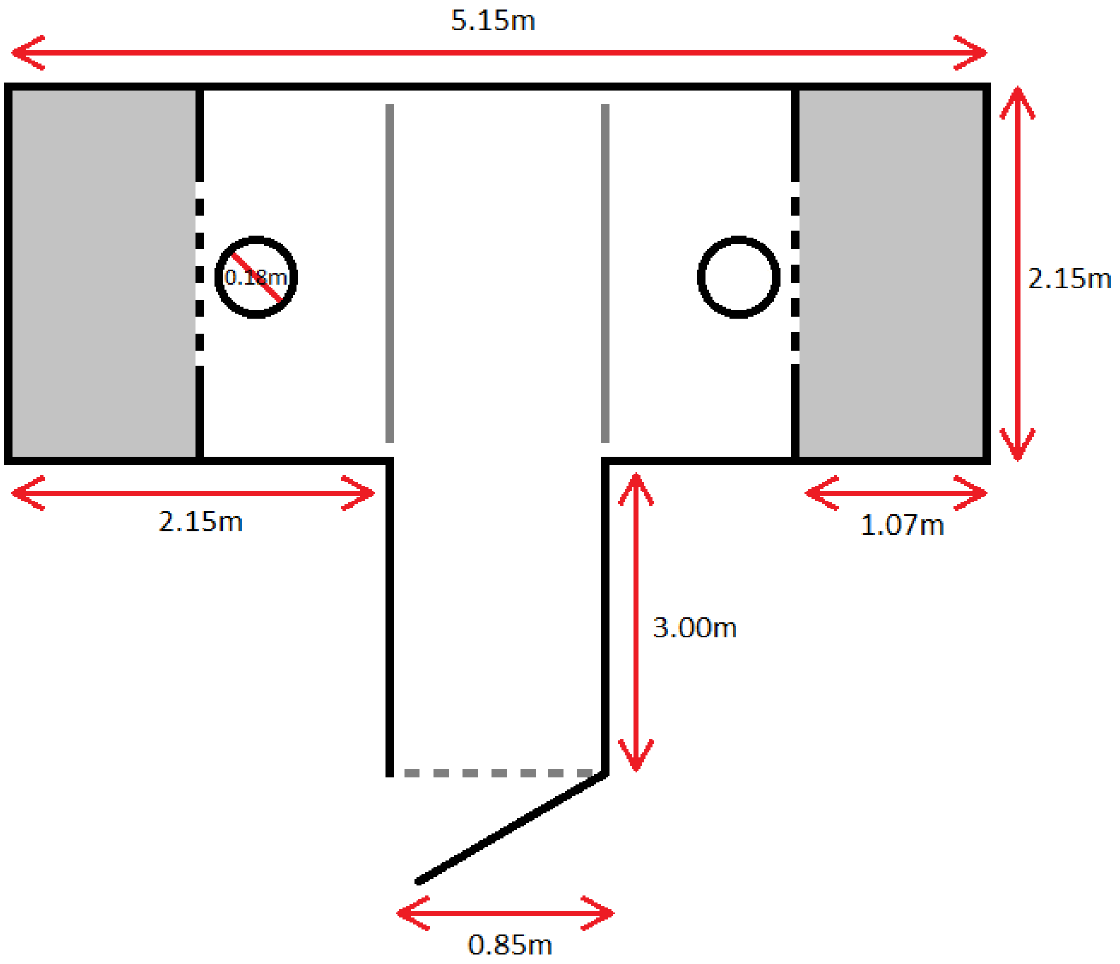

Supplementary Figure 2 – The T-maze used for the Social Preference, Novel Object, and Puzzle Box tests. Grey areas represent the holding pens for pairs of stimulus pigs during the social preference test. Solid Grey lines indicate division of branches from the central branch and were not visible to the test individual during the trial. Black dashed lines indicate the location of the grated divider used to separate test pigs from stimulus pigs. The dashed grey line indicates the location of the gate when in the closed position. Rewarded food bowls (Brown Lettered Dog Bowl – 18cm Diameter, RSPCA) were only present during the social preference test and contained crushed banana and strawberry jam.

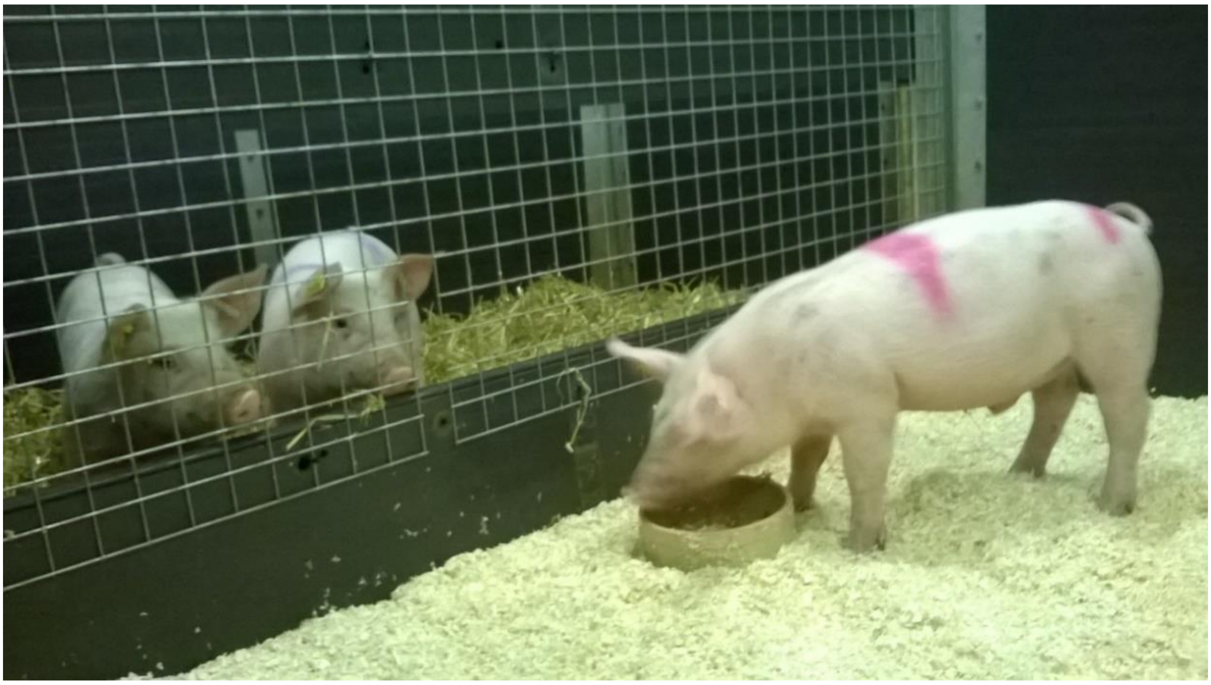

Supplementary Figure 3 – The division grid used to separate stimulus pigs from the test pig during the social preference test (Photo Credit: Simon Turner).

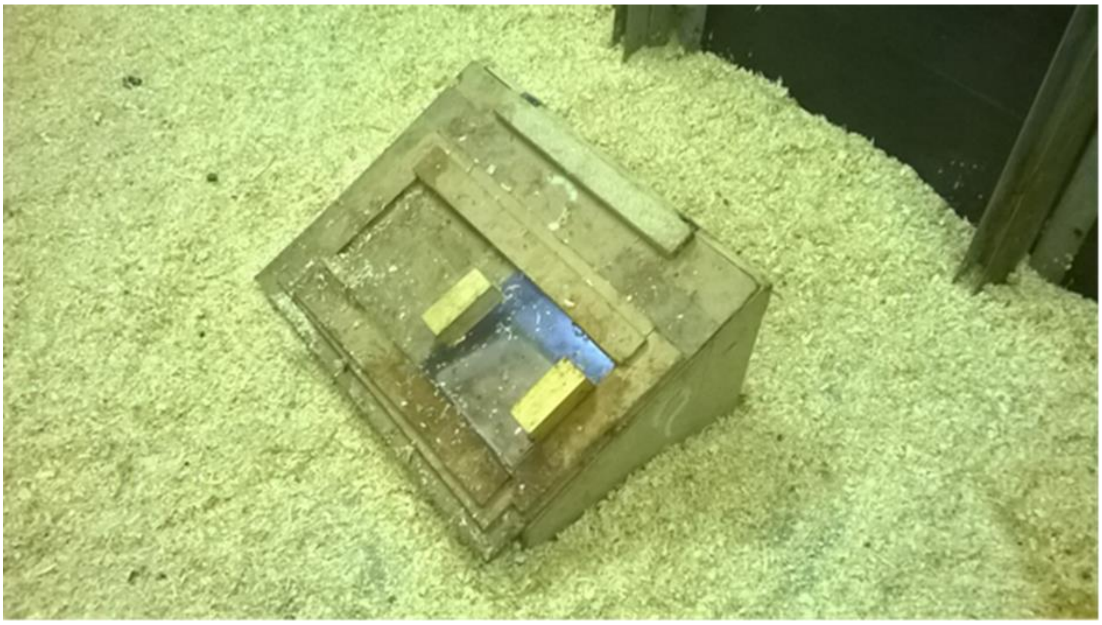

Supplementary Figure 4 – Puzzle box with sliding door in the closed position. Dimensions: 30 x 40 x 65 cm (Photo Credit: Simon Turner).

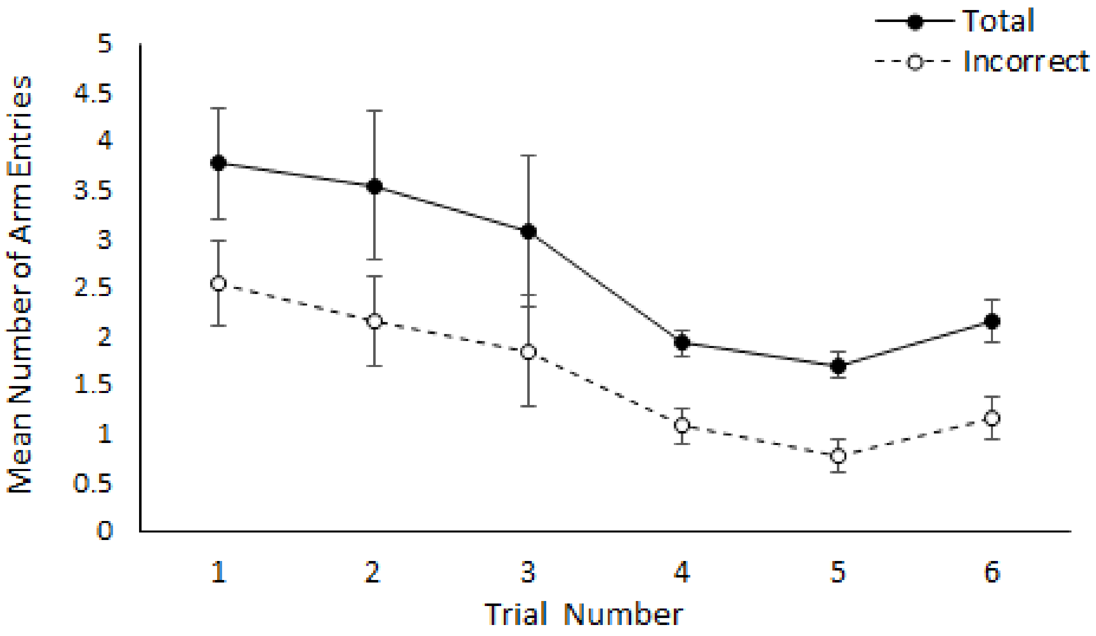

Supplementary Figure 5 – The mean number of total (solid black line) and incorrect (dashed black line) entries made into either arm of the T-maze over the course of 6 reversal learning trials. Error bars represent the standard error of the mean.

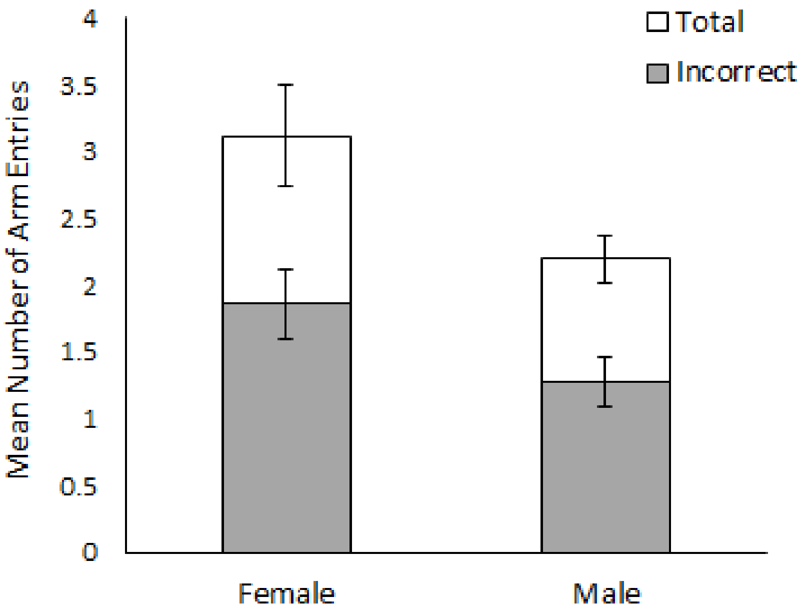

Supplementary Figure 6 – The mean number of total (white and grey portion of bar) and incorrect (grey portion of bar) entries made into either arm of the T-maze during a reversal learning trial for both females and males. Error bars represent the standard error of the mean.

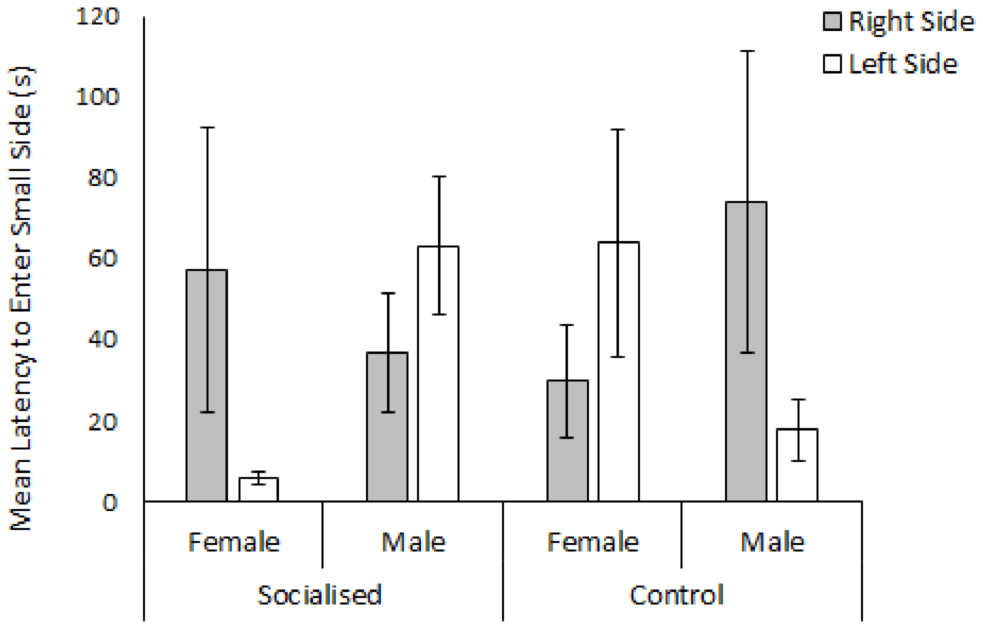

Supplementary Figure 7 – The mean latency (s) of socialised females, socialised males, control females, and control females to enter into the side of the social preference T-maze containing the small stimulus pigs when the small stimulus pigs were located on the right (grey bars) and left (white bars) side of the maze. Error bars represent the standard error of the mean.

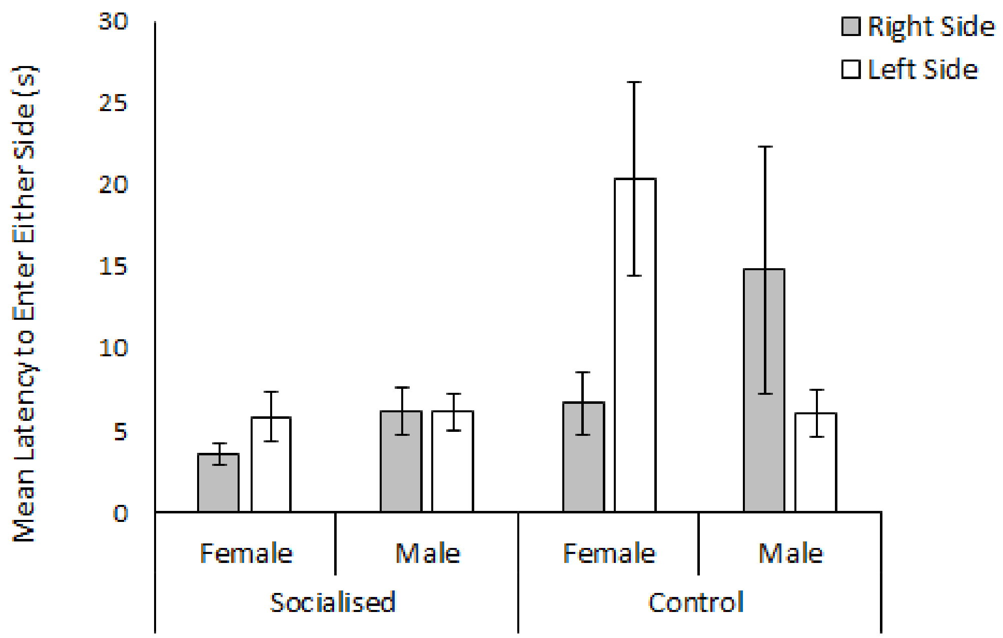

Supplementary Figure 8 – The mean latency (s) of socialised females, socialised males, control females, and control females to enter into either side of the social preference T-maze when the small stimulus pigs were located on the right (grey bars) and left (white bars) side of the maze. Error bars represent the standard error of the mean.

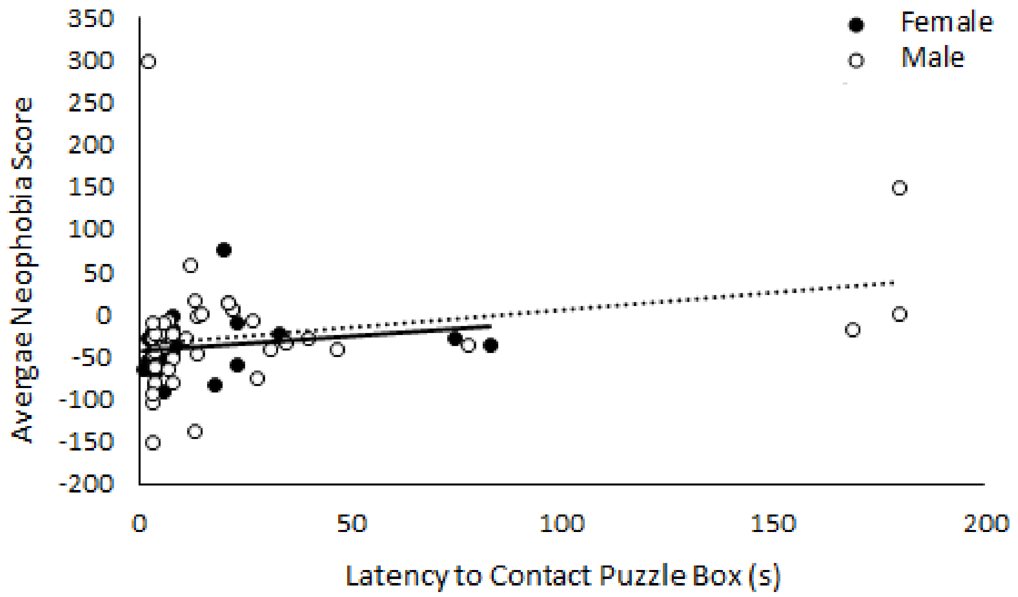

Supplementary Figure 9 – The relationship between average neophobia scores and latency to contact the puzzle box (s) presented for both females (black circles and solid black trendline) and males (white circles with dashed black trendline).

Supplementary Table 1– Model outputs for all general and generalised linear mixed effects models pertaining to the food reward test. Test statistics (*χ^2, D.F.* and *p*-values) were extracted for all factors and interactions retained within the model by means of a Wald's test. ':' indicates an interaction effect between 2 or more fixed factors. [a] indicates an outcome variable that was square root (+0.0001) transformed. [b] indicates an outcome variable was log transformed. [*] indicates fixed factors of interest that were not removed from the model regardless of significance. **Bold** *P*-values indicate significance (α=0.05). Marginal R^2 value (which represents the variance explained by the combined fixed effects within a model) and conditional R^2 value (which represents the variance explained by both the fixed and random effects within a model) are given beneath each model.

	χ^2	D.F.	*P*
**Trial Duration**			
Trial[*]	6.5951	11	0.83087
Treatment[*]	0.3137	1	0.57539
Sex[*]	0.0366	1	0.84830
Treatment:Sex	3.3715	1	0.06633
			**Marginal R^2** = 0.0291857
			**Conditional R^2** = 0.494112
**Total Number of Arm Entries[a]**			
Trial[*]	6.4976	11	0.83819
Treatment[*]	0.3142	1	0.57508
Sex[*]	0.5901	1	0.44238
Trial:Sex	20.4358	11	**0.03971**
			**Marginal R^2** = 0.04164434
			**Conditional R^2** = 0.2947142
**Total Number of Incorrect Entries[a]**			
Trial[*]	3.5031	11	0.98227
Treatment[*]	1.1062	1	0.29291
Sex[*]	1.1504	1	0.28347
Reward_Side	1.6488	1	0.19912
Trial:Treatment	18.1312	11	0.07859
Trial:Sex	14.8104	11	0.19134
Treatment:Sex	0.1420	1	0.70629
Trial:Reward_Side	9.4173	11	0.58343
Treatment:Reward_Side	0.8462	1	0.35762

Sex:Reward_Side	0.0760	1	0.78275
Trial:Sex:Reward_Side	17.2927	11	0.09951
			**Marginal R^2** = 0.09754011
			**Conditional R^2** = 0.4340249
**Total Number of Correct Entries[a]**			
Trial*	17.8386	11	0.08540
Treatment*	0.0111	1	0.91599
Sex*	0.3356	1	0.56236
Reward_Side	0.1292	1	0.71922
Trial:Treatment	10.9475	11	0.44768
Trial:Sex	15.8507	11	0.14676
Treatment:Sex	0.0130	1	0.90911
Trial:Reward_Side	3.9570	11	0.97116
Treatment:Reward_Side	2.8050	1	0.09397
Sex:Reward_Side	2.3164	1	0.12801
Trial:Treatment:Sex	10.7061	11	0.46820
Trial:Treatment:Reward_Side	10.7455	11	0.46483
Trial:Sex:Reward_Side	13.8302	11	0.24253
Treatment:Sex:Reward_Side	4.6733	1	**0.03064**
Trial:Treatment:Sex:Reward_Side	18.8831	11	0.06322
			**Marginal R^2** = 0.1728744
			**Conditional R^2** = 0.2948578
**Likelihood of Success**			
Trial*	13.4189	11	0.26683
Sex*	0.3119	1	0.57652
Treatment*	0.0800	1	0.77728
Reward_Side	0.0349	1	0.85190
Trial:Sex	18.0286	11	0.08092
Trial:Treatment	13.4968	11	0.26210
Sex:Treatment	0.1023	1	0.74905
Trial:Reward_Side	14.9465	11	0.18496
Sex:Reward_Side	0.7587	1	0.38373
Treatment:Reward_Side	0.1973	1	0.65693
Sex:Treatment:Reward_Side	3.9594	1	**0.04661**
			**Marginal R^2** = 0.1096047
			**Conditional R^2** = 0.2362543
**Likelihood of Progression to Reversal Learning Test**			
Sex*	0.5006	1	0.4792
Treatment*	0.3636	1	0.5465
			**Marginal R^2 =** 0.03017844
			**Conditional R^2** = 0.7708656

Supplementary Table 2– Model outputs for all general and generalised linear mixed effects models pertaining to the reversal learning test. Test statistics (χ^2, *D.F.* and *p*-values) were extracted for all factors and interactions retained within the model by means of a Wald's test. ':' indicates an interaction effect between 2 or more fixed factors. [a] indicates an outcome variable that was square root (+0.0001) transformed. [b] indicates an outcome variable was log transformed. [*] indicates fixed factors of interest that were not removed from the model regardless of significance. **Bold** *p*-values indicate significance (α=0.05). Marginal R^2 value (which represents the variance explained by the combined fixed effects within a model) and conditional R^2 value (which represents the variance explained by both the fixed and random effects within a model) are given beneath each model.

	χ^2	D.F.	*P*
**Trial Duration**			
Trial[*]	42.5727	5	**<0.001**
Treatment[*]	0.0007	1	0.979088
Sex[*]	1.1378	1	0.286110
Trial:Treatment	16.9284	5	**0.004638**
			**Marginal R^2** = 0.2622558
			**Conditional R^2** = 0.7230358
**Total Number of Arm Entries[a]**			
Trial[*]	23.1085	5	0.0003218
Sex[*]	5.2187	1	0.0223450
Treatment[*]	0.8384	1	0.3598564
			**Marginal R^2** = 0.2514106
			**Conditional R^2** = 0.3954336
**Total Number of Incorrect Entries[a]**			
Trial[*]	27.9150	5	**<0.001**
Sex[*]	3.9350	1	**0.04729**
Treatment[*]	0.9583	1	0.32762
			**Marginal R^2** = 0.2436463
			**Conditional R^2** = 0.4928805
**Total Number of Correct Entries[a]**			
Trial[*]	5.1063	5	0.40305
Sex[*]	2.8495	1	0.09140
Treatment[*]	0.3427	1	0.55830
Sex:Treatment	2.9502	1	0.08587
			**Marginal R^2** = 0.1405884
			**Conditional R^2** = 0.1996627

Likelihood of Success			
Trial*	2.4303	5	0.7870
Sex*	0.9851	1	0.3209
Treatment*	1.1782	1	0.2777

Marginal R^2 = 0.9846168
Conditional R^2 = 0.9946897

Supplementary Table 3– Model outputs for all general and generalised linear mixed effects models pertaining to the social preference test. Test statistics (χ^2, *D.F.* and *p*-values) were extracted for all factors and interactions retained within the model by means of a Wald's test. ':' indicates an interaction effect between 2 or more fixed factors. [a] indicates an outcome variable that was square root (+0.0001) transformed. [b] indicates an outcome variable was log transformed. [*] indicates fixed factors of interest that were not removed from the model regardless of significance. **Bold** *p*-values indicate significance (α=0.05). Marginal R^2 value (which represents the variance explained by the combined fixed effects within a model) and conditional R^2 value (which represents the variance explained by both the fixed and random effects within a model) are given beneath each model.

	χ^2	D.F.	*P*
**Percentage of Time in Side**			
Side[*]	42.5727	5	**<0.001**
Treatment[*]	0.0007	1	0.979088
Sex[*]	1.1378	1	0.286110
Side:Treatment	16.9284	5	**0.004638**
			**Marginal R^2** = 0.3146421
			**Conditional R^2** = 0.3146421
**Latency to Enter the Small Side[b]**			
Treatment[*]	0.6063	1	0.43618
Sex[*]	3.1993	1	0.07367
Side_of_Small	0.1545	1	0.69428
Treatment:Sex	1.4835	1	0.22323
Treatment:Side_of_Small	0.2182	1	0.64041
Sex:Side_of_Small	0.0570	1	0.81131
Treatment:Sex:Side_of_Small	6.6238	1	**0.016006**
			**Marginal R^2** = 0.1926156
			**Conditional R^2** = 0.2102219
**Likelihood of Entering into the Small Side First**			
Treatment[*]	0.8983	1	0.3432
Sex[*]	2.5040	1	0.1136
			**Marginal R^2** = 0.07214691
			**Conditional R^2** = 0.07214691
**Latency to Enter into Either Side[b]**			
Treatment[*]	12.1581	1	**<0.001**

Sex*	0.2519	1	0.6157439
Side_of_Small	3.5746	1	0.0586682
Treatment:Sex	1.0994	1	0.2943927
Treatment:Side_of_Small	0.0142	1	0.9051523
Sex:Side_of_Small	9.1637	1	**0.0024686**
Treatment:Sex:Side_of_Small	4.3947	1	**0.0360518**

Marginal R^2 = 0.3229871
Conditional R^2 = 0.4260179

Supplementary Table 4– Model outputs for all general and generalised linear mixed effects models pertaining to the novel object and puzzle box tests. Test statistics (χ^2, *D.F.* and *p*-values) were extracted for all factors and interactions retained within the model by means of a Wald's test. ':' indicates an interaction effect between 2 or more fixed factors. [a] indicates an outcome variable that was square root (+0.0001) transformed. [b] indicates an outcome variable was log transformed. [*] indicates fixed factors of interest that were not removed from the model regardless of significance. **Bold** *p*-values indicate significance (α=0.05). Marginal R^2 value (which represents the variance explained by the combined fixed effects within a model) and conditional R^2 value (which represents the variance explained by both the fixed and random effects within a model) are given beneath each model.

	χ^2	D.F.	*P*
**Average Neophobia Score**			
Treatment*	0.3303	1	0.5655
Sex*	0.5961	1	0.4401
			**Marginal R^2** = 0.01574003
			**Conditional R^2** = 0.8101406
**Latency to Contact Puzzle Box[b]**			
Treatment*	1.9335	1	0.1643728
Sex*	3.6135	1	0.0573121
Attempt	13.1401	1	**0.0002890**
Average_Neophobia*	13.2381	1	**0.0002743**
Treatment:Sex	0.7722	1	0.3795358
Treatment:Attempt	1.4669	1	0.2258297
Sex:Attempt	0.7756	1	0.3784899
Treatment:Average_Neophobia	0.9289	1	0.3351482
Sex:Average_Neophobia	4.0699	1	**0.0436526**
Attempt:Average_Neophobia	11.8938	1	**0.0005632**
Treatment:Sex:Attempt	0.8079	1	0.3687376
Treatment:Sex:Average_Neophobia	<0.0001	1	0.9960004
Treatment:Attempt:Average_Neophobia	1.7233	1	0.9055473
Sex:Attempt:Average_Neophobia	17.233	1	0.1892735
Treatment:Sex:Attempt:Average_Neophobia	5.3177	1	**0.0211831**
			**Marginal R^2** = 0.2565817
			**Conditional R^2** = 0.4482059

**Percentage of Attempt Spent in Contact with Puzzle Box**[b]			
Treatment*	0.1944	1	0.65926
Sex*	1.2075	1	0.27183
Attempt*	5.5489	1	**0.01559**
Average_Neophobia*	2.7462	1	0.09749
Treatment:Average_Neophobia			
			**Marginal R^2** = 0.05735927
			**Conditional R^2** = 0.6068549
**Time to Solve Puzzle**[b]			
Treatment*	0.0134	1	0.907933
Sex*	1.2262	1	0.268140
Attempt*	6.7772	1	**0.009233**
Average_Neophobia*	2.8101	1	0.093672
			**Marginal R^2** =0.04656172
			**Conditional R^2** =0.4339058
**Likelihood of Solving Puzzle**			
Treatment*	0.1172	1	0.73213
Sex*	0.1986	1	0.65585
Test*	0.0055	1	0.94093
Average_Neophobia*	3.0168	1	0.08241
Treatment:Average_Neophobia	1.5131	1	0.21867
			**Marginal R^2** = 0.07658225
			**Conditional R^2** =0.7399695

Supplementary Table 5 – β values and standard error (S.E.) pertaining to the analysis of the food reward test. β values and S.E. were calculated using linear regressions and included all fixed factors remaining in the final general and generalised linear mixed effects models. [a] indicates an outcome variable that was square root (+0.0001) transformed. [b] indicates an outcome variable was log transformed.

	β	± S.E.
**Trial Duration**		
**Trial**		
Trial B	-13.453	14.053
Trial C	-13.294	16.698
Trial D	-12.555	16.530
Trial E	-1.828	16.614
Trial F	-19.577	16.531
Trial G	-13.832	16.449
Trial H	-10.493	16.449
Trial I	-4.087	16.612
Trial J	-11.768	16.449
Trial K	-10.619	16.449
Trial L	3.360	16.449
**Treatment**		
Socialised	-1.092	16.449
**Sex**		
Male	9.531	9.636
**Treatment:Sex**		
Socialised:Male	-28.462	13.093
**Total Number of Arm Entries[a]**		
**Trial**		
Trial B	-0.060794	0.146411
Trial C	-0.002449	0.189913
Trial D	0.092996	0.188114
Trial E	0.264286	0.188114
Trial F	0.076609	0.188114
Trial G	0.198896	0.188114
Trial H	0.152520	0.188114
Trial I	0.113307	0.191912
Trial J	-0.048067	0.188114
Trial K	0.057360	0.188114
Trial L	-0.096227	0.188114
**Treatment**		
Socialised	0.059604	0.051042
**Sex**		
Male	0.032618	0.192071

**Trial:Sex**		
Trial B:Male	-0.017268	0.261269
Trial C:Male	-0.087984	0.258641
Trial D:Male	-0.257221	0.259971
Trial E:Male	-0.549979	0.258633
Trial F:Male	-0.072583	0.257421
Trial G:Male	-0.217281	0.257421
Trial H:Male	-0.204199	0.260203
Trial I:Male	0.009850	0.257421
Trial J:Male	0.094724	0.257421
Trial K:Male	-0.025378	0.257421
Trial L:Male	0.280561	0.257421
**Total Number of Incorrect Entries[a]**		
**Trial**		
Trial B	-0.168404	0362632
Trial C	0.046554	0.353298
Trial D	-0.070342	0.353649
Trial E	0.316116	0.353426
Trial F	-0.169985	0.353017
Trial G	0.029842	0.353017
Trial H	0.084187	0.356674
Trial I	-0.111549	0.353017
Trial J	-0.153762	0.353017
Trial K	0.235194	0.353017
Trial L	-0.021992	0.353017
**Treatment**		
Socialised	0.068033	0.248995
**Sex**		
Male	0.195365	0.319913
**Reward Side**		
Right	-0.371681	0.350456
**Trial:Treatment**		
Trial B:Socialised	-0.244277	0.316449
Trial C:Socialised	-0.507131	0.313343
Trial D:Socialised	0.111745	0.315244
Trial E:Socialised	-0.030358	0.313969
Trial F:Socialised	0.162874	0.311811
Trial G:Socialised	0.354895	0.311811
Trial H:Socialised	0.021994	0.314379
Trial I:Socialised	0.001984	0.311811
Trial J:Socialised	0.028366	0.311811
Trial K:Socialised	-0.160446	0.311811
Trial L:Socialised	0.019322	0.311811
**Trial:Sex**		
Trial B:Male	-0.049002	0.430501
Trial C:Male	-0.033297	0.425601
Trial D:Male	-0.473053	0.430137
Trial E:Male	-0.750378	0.421931
Trial F:Male	-0.450229	0.421931

Trial G:Male	-0.560031	0.421931
Trial H:Male	-0.586908	0.427272
Trial I:Male	-0.162565	0.421931
Trial J:Male	-0.060467	0.421931
Trial K:Male	-0.438941	0.421931
Trial L:Male	-0.327943	0.421931
**Treatment:Sex**		
Socialised:Male	0.037079	0.122695
**Trial:Reward_Side**		
Trial B:Right	0.651383	0.453369
Trial C:Right	0.490060	0.449693
Trial D:Right	0.203801	0.449812
Trial E:Right	-0.254559	0.449722
Trial F:Right	-0.091405	0.449598
Trial G:Right	-0.270240	0.449598
Trial H:Right	0.026885	0.459498
Trial I:Right	0.349201	0.449598
Trial J:Right	0.115258	0.449598
Trial K:Right	-0.227609	0.449598
Trial L:Right	-0.291758	0.449598
**Treatment:Reward_Side**		
Socialised:Right	0.283951	0.122619
**Sex:Reward_Side**		
Male:Right	-0.314528	0.458883
**Trial:Sex:Treatment**		
Trial B:Male:Right	-0.214467	0.621416
Trial C:Male:Right	0.014915	0.616280
Trial D:Male:Right	0.358855	0.620553
Trial E:Male:Right	0.680652	0.617720
Trial F:Male:Right	0.864193	0.612834
Trial G:Male:Right	0.775716	0.612834
Trial H:Male:Right	0.483542	0.620156
Trial I:Male:Right	0.106432	0.612834
Trial J:Male:Right	0.337945	0.612834
Trial K:Male:Right	0.835095	0.612834
Trial L:Male:Right	1.209852	0.612834
**Total Number of Correct Entries[a]**		
**Trial**		
Trial B	-0.185489	0.311146
Trial C	-0.063484	0.299400
Trial D	0.147836	0.299400
Trial E	0.074583	0.299400
Trial F	-0.063484	0.299400
Trial G	-0.063484	0.299400
Trial H	0.331128	0.299400
Trial I	0.005549	0.299400
Trial J	0.005549	0.299400
Trial K	0.314499	0.299400
Trial L	-0.106487	0.299400

**Treatment**		
Treatment	0.021611	0.327977
**Sex**		
Male	-0.377439	0.299400
**Reward_Side**		
Right	-0.185489	0.401688
**Trial:Treatment**		
Trial B:Socialised	-0.073215	0.440027
Trial C:Socialised	-0.058791	0.431801
Trial D:Socialised	-0.125690	0.431801
Trial E:Socialised	0.293965	0.431801
Trial F:Socialised	0.515576	0.431801
Trial G:Socialised	0.104904	0.431801
Trial H:Socialised	-0.331128	0.444082
Trial I:Socialised	-0.066243	0.431801
Trial J:Socialised	-0.608669	0.431801
Trial K:Socialised	-0.636769	0.431801
Trial L:Socialised	0.265062	0.431801
**Trial:Sex**		
Trial B:Male	0.047422	0.410517
Trial C:Male	-0.005549	0.401688
Trial D:Male	-0.071056	0.410517
Trial E:Male	-0.106332	0.395260
Trial F:Male	0.314605	0.395260
Trial G:Male	0.373777	0.373777
Trial H:Male	-0.139178	0.395260
Trial I:Male	0.350148	0.395260
Trial J:Male	0.454724	0.395260
Trial K:Male	-0.004206	0.395260
Trial L:Male	0.462184	0.395260
**Treatment:Sex**		
Socialised:Male	0.308406	0.423416
**Trial:Reward_Side**		
Trial B:Right	0.518814	0.525445
Trial C:Right	0.201550	0.518577
Trial D:Right	-0.147836	0.518577
Trial E:Right	0.063484	0.518577
Trial F:Right	0.613563	0.518577
Trial G:Right	0.612057	0.518577
Trial H:Right	-0.327820	0.518577
Trial I:Right	-0.091556	0.518577
Trial J:Right	-0.002241	0.518577
Trial K:Right	-0.506449	0.518577
Trial L:Right	-0.085463	0.518577
**Treatment:Reward_Side**		
Socialised:Right	0.037560	0.495854
**Sex:Reward_Side**		
Male:Right	0.606685	0.490139
**Trial:Treatment:Sex**		
Trial B:Socialised:Male	0.308911	0.580559

Trial C:Socialised:Male	-0.010242	0.574349
Trial D:Socialised:Male	0.077506	0.580559
Trial E:Socialised:Male	-0.262215	0.569873
Trial F:Socialised:Male	-0.835731	0.569873
Trial G:Socialised:Male	-0.225251	0.569873
Trial H:Socialised:Male	0.289779	0.579234
Trial I:Socialised:Male	-0.114478	0.569873
Trial J:Socialised:Male	0.298997	0.569873
Trial K:Socialised:Male	0.310414	0.569873
Trial L:Socialised:Male	-0.732796	0.569873
**Trial:Treatment:Reward_Side**		
Trial B:Socialised:Right	-0.383265	0.653868
Trial C:Socialised:Right	-0.087401	0.648361
Trial D:Socialised:Right	0.148958	0.648361
Trial E:Socialised:Right	-0.196734	0.648361
Trial F:Socialised:Right	-1.043491	0.648361
Trial G:Socialised:Right	-0.402432	0.648361
Trial H:Socialised:Right	0.326075	0.658875
Trial I:Socialised:Right	0.331546	0.648361
Trial J:Socialised:Right	0.482206	0.648361
Trial K:Socialised:Right	0.953636	0.648361
Trial L:Socialised:Right	-0.150244	0.648361
**Trial:Sex:Reward_Side**		
Trial B:Male:Right	-0.297908	0.658681
Trial C:Male:Right	-0.126067	0.647702
Trial D:Male:Right	-0.089156	0.653214
Trial E:Male:Right	-0.356955	0.643736
Trial F:Male:Right	-0.696886	0.643736
Trial G:Male:Right	-1.109505	0.643736
Trial H:Male:Right	0.455366	0.643736
Trial I:Male:Right	0.024910	0.643736
Trial J:Male:Right	-0.518958	0.643736
Trial K:Male:Right	0.324611	0.643736
Trial L:Male:Right	0.266262	0.643736
**Treatment:Sex:Reward_Side**		
Socialised:Male:Right	-0.596823	0.643736
**Trial:Treatment:Sex:Reward_Side**		
Trial B:Socialised:Male:Right	-0.133281	0.868236
Trial C:Socialised:Male:Right	0.149984	0.859937
Trial D:Socialised:Male:Right	0.205844	0.864097
Trial E:Socialised:Male:Right	0.490205	0.863207
Trial F:Socialised:Male:Right	1.690262	0.856954
Trial G:Socialised:Male:Right	0.256340	0.856954
Trial H:Socialised:Male:Right	-0.802232	0.856954
Trial I:Socialised:Male:Right	-0.357036	0.856954
Trial J:Socialised:Male:Right	-0.111608	0.856954
Trial K:Socialised:Male:Right	-0.755737	0.856954
Trial L:Socialised:Male:Right	0.164323	0.856954

**Likelihood of Success**		
**Trial**		
Trial B	0.09841	0.23482
Trial C	0.15054	0.22987
Trial D	0.09364	0.23092
Trial E	-0.07875	0.23023
Trial F	0.34541	0.22898
Trial G	0.32810	0.22898
Trial H	0.29393	0.23018
Trial I	0.28914	0.22898
Trial J	0.35602	0.22898
Trial K	0.16613	0.22898
Trial L	0.32531	0.22898
**Sex**		
Male	-0.18734	0.17656
**Treatment**		
Socialised	-0.18723	0.18009
**Reward_Side**		
Right	0.05387	0.18626
**Trial:Sex**		
Trial B:Male	0.20193	0.22004
Trial C:Male	0.09559	0.21752
Trial D:Male	0.31447	0.21833
Trial E:Male	0.20424	0.21837
Trial F:Male	0.01712	0.21684
Trial G:Male	0.20322	0.21684
Trial H:Male	0.23780	0.21872
Trial I:Male	0.07658	0.21684
Trial J:Male	-0.13219	0.21684
Trial K:Male	0.04858	0.21684
Trial L:Male	-0.21324	0.21684
**Trial:Treatment**		
Trial B:Socialised	0.26687	0.22174
Trial C:Socialised	0.14385	0.21885
Trial D:Socialised	-0.10753	0.21974
Trial E:Socialised	0.11659	0.21914
Trial F:Socialised	-0.04225	0.21810
Trial G:Socialised	-0.22443	0.21810
Trial H:Socialised	0.03403	0.21965
Trial I:Socialised	0.10115	0.21810
Trial J:Socialised	0.11103	0.21810
Trial K:Socialised	0.12989	0.21810
Trial L:Socialised	0.09203	0.21810
**Treatment:Sex**		
Socialised:Male	0.24755	0.11787
**Trial:Reward_Side**		
Trial B:Right	-0.47605	0.21857
Trial C:Right	-0.55164	0.21602
Trial D:Right	-0.31596	0.21686
Trial E:Right	-0.08035	0.21633

Trial F:Right	-0.29134	0.21532
Trial G:Right	-0.29563	0.21532
Trial H:Right	-0.37316	0.21758
Trial I:Right	-0.35080	0.21532
Trial J:Right	-0.32386	0.21532
Trial K:Right	-0.23190	0.21532
Trial L:Right	-0.24281	0.21532
**Sex:Reward_Side**		
Male:Right	0.40876	0.12912
**Treatment:Reward_Side**		
Socialised:Right	0.27641	0.12891
**Treatment:Sex:Reward_Side**		
Socialised:Male:Right	-0.72631	0.17449
**Likelihood of Progression to Reversal Learning Test**		
**Treatment**		
Socialised	-0.05417	0.13872
**Sex**		
Male	-0.10597	0.13847

Supplementary Table 6 – β values and standard error (S.E.) pertaining to the analysis of the reversal learning test. β values and S.E. were calculated using linear regressions and included all fixed factors remaining in the final general and generalised linear mixed effects models. [a] indicates an outcome variable that was square root (+0.0001) transformed. [b] indicates an outcome variable was log transformed.

	β	± S.E.
**Trial Duration**		
**Trial**		
Trial B	-27.86	29.35
Trial C	-85.71	29.35
Trial D	-95.00	29.35
Trial E	-98.86	29.35
Trial F	-92.00	29.35
**Treatment**		
Socialised	-38.79	30.70
**Sex**		
Male	-28.51	12.84
**Trial:Treatment**		
Trial B:Socialised	-9.81	43.21
Trial C:Socialised	75.88	43.21
Trial D:Socialised	57.00	43.21
Trial E:Socialised	58.02	43.21
Trial F:Socialised	55.83	43.21
**Total Number of Arm Entries[a]**		
**Trial**		
Trial B	-0.08278	0.16389
Trial C	-0.21795	0.16389
Trial D	-0.50258	0.16389
Trial E	-0.59075	0.16389
Trial F	0.43307	0.16389
Treatment		
Socialised	0.09771	0.122
Sex		
Male	0.09771	-2.408
**Total Number of Incorrect Entries[a]**		
**Trial**		
Trial B	-0.14239	0.15661
Trial C	-0.27092	0.18888
Trial D	-0.54019	0.18888
Trial E	-0.79311	0.18888
Trial F	-0.55263	0.18888
**Treatment**		
Socialised	0.07263	0.11261

**Sex**		
Male	-0.22139	0.11261
**Total Number of Correct Entries[a]**		
**Trial**		
Trial B	0.02333	0.13047
Trial C	0.03305	0.13047
Trial D	-0.19619	0.13047
Trial E	-0.12003	0.13047
Trial F	-0.04388	0.13047
**Treatment**		
Socialised	-0.19080	0.10371
**Sex**		
Male	-0.29097	0.10371
**Treatment:Sex**		
Socialised:Male	0.31455	0.15680
**Likelihood of Success**		
**Trial**		
Trial B	3.557×10^{-17}	0.1053
Trial C	2.737×10^{-17}	0.1503
Trial D	0.07692	0.1503
Trial E	0.3077	0.1503
Trial F	0.1538	0.1503
**Treatment**		
Socialised	-0.09896	0.06277
**Sex**		
Male	0.06771	0.06771

Supplementary Table 7 – β values and standard error (S.E.) pertaining to the analysis of the social preference test. β values and S.E. were calculated using linear regressions and included all fixed factors remaining in the final general and generalised linear mixed effects models. [a] indicates an outcome variable that was square root (+0.0001) transformed. [b] indicates an outcome variable was log transformed.

	β	± S.E.
**Percentage of Time in Side**		
**Side**		
Middle	-11.459	6.573
Small	20.485	6.573
**Treatment**		
Socialised	15.338	6.446
**Side:Treatment**		
Middle:Socialised	-22.899	9.115
Small:Socialised	-23.110	9.115
**Latency to Enter the Small Side[b]**		
**Treatment**		
Socialised	-1.9308	0.7640
**Sex**		
Male	-0.9253	0.8541
**Side_of_Small**		
Right	-1.0396	0.6974
**Treatment:Sex**		
Socialised:Male	2.9747	1.1027
**Treatment:Side_of_Small**		
Socialised:Right	2.3635	1.1027
**Sex:Side_of_Small**		
Male:Right	1.8663	1.1288
**Treatment:Sex:Side_of_Small**		
Socialised:Male:Right	-3.9709	1.5648
**Likelihood of Entering into the Small Side First**		
**Treatment**		
Socialised	0.1361	0.1460
**Sex**		
Male	-0.2327	0.1459
**Latency to Enter Either Side[b]**		
**Treatment**		
Socialised	-1.1783	0.3575
**Sex**		
Male	-1.0950	0.3997
**Side_of_Small**		
Right	-1.1345	0.3264

**Treatment:Sex**		
Socialised:Male	1.1803	0.5160
**Treatment:Side_of_Small**		
Socialised:Right	0.7140	0.5160
**Sex:Side_of_Small**		
Male:Right	1.7616	0.5283
**Treatment:Sex:Side_of_Small**		
Socialised:Male:Right	-1.3976	0.7323

Supplementary Table 8 – β values and standard error (S.E.) pertaining to the analysis of the novel object and puzzle box tests. β values and S.E. were calculated using linear regressions and included all fixed factors remaining in the final general and generalised linear mixed effects models. [a] indicates an outcome variable that was square root (+0.0001) transformed. [b] indicates an outcome variable was log transformed.

	β	± S.E.
**Average Neophobia Score**		
**Treatment**		
Socialised	2.650	10.449
**Sex**		
Male	12.338	10.440
**Latency to Contact Puzzle[b]**		
**Treatment**		
Socialised	2.289256	1.046546
**Sex**		
Male	1.635388	0.798620
**Attempt**		
Attempt	0.310889	0.405422
**Average_Neophobia**		
Average_Neophobia	-0.001229	0.013354
**Treatment:Sex**		
Socialised:Male	-2.144204	1.215269
**Treatment:Attempt**		
Male:Attempt	-1.397492	0.650033
**Sex:Attempt**		
Male:Attempt	-1.146029	0.501229
**Treatment:Average_Neophobia**		
Socialised:Average_Neophobia	0.036577	0.021621
**Sex:Average_Neophobia**		
Male:Average_Neophobia	0.025089	0.015376
**Attempt:Average_Neophobia**		
Attempt:Average_Neophobia	0.008808	0.008426
**Treatment:Sex:Attempt**		
Socialised:Male:	1.466501	0.757828
**Treatment:Sex:Average_Neophobia**		
Socialised:Male:Average_Neophobia	-0.046633	0.023481
**Treatment:Attempt:Average_Neophobia**		
Socialised:Attempt:Average_Neophobia	-0.023603	0.013530
**Sex:Attempt:Average_Neophobia**		
Male:Attempt:Average_Neophobia	-0.020853	0.009708
**Treatment:Sex:Attempt:Average_Neophobia**		
Socialised:Male:Attempt:Average_Neophobia	0.028664	0.014718

**Percentage of Attempt Spent in Contact with Puzzle Box**[b]		
**Treatment**		
Socialised	0.306552	0.202501
**Sex**		
Male	0.235904	0.162673
**Attempt**		
Attempt	-0.237031	0.161635
**Average_Neophobia**		
Average_Neophobia	-0.006298	0.002962
**Treatment:Average_Neophobia**		
Socialised:Average_Neophobia	0.005922	0.003534
**Time to Solve Puzzle**[b]		
**Treatment**		
Socialised	-0.041315	0.179393
**Sex**		
Male	-0.251650	0.180452
**Attempt**		
Attempt	-0.376347	0.179331
**Average Neophobia**		
	0.003156	0.001806
**Treatment:Average_Neophobia**		
Socialised:Right		
**Likelihood of Solving Puzzle**		
**Treatment**		
Socialised	0.0348494	0.0731602
**Sex**		
Male	0.0522155	0.0735918
**Attempt**		
Attempt	0.0007221	0.0731347
**Average_Neophobia**		
Average Neophobia	-0.0017396	0.0007365
